# l-Ascorbic Acid Treatment of Electrochemical Graphene Nanosheets: Reduction Optimization and Application for De-Icing, Water Uptake Prevention, and Corrosion Resistance

**DOI:** 10.1021/acsami.2c22854

**Published:** 2023-04-26

**Authors:** Markus Ostermann, Pierluigi Bilotto, Martin Kadlec, Jürgen Schodl, Jiri Duchoslav, Michael Stöger-Pollach, Peter Lieberzeit, Markus Valtiner

**Affiliations:** † CEST GmbH, Centre for Electrochemical Surface Technology, A-2700 Wiener Neustadt, Austria; ‡ Institute of Physical Chemistry, University of Vienna, A-1090 Vienna, Austria; ¶ VZLU − Czech Aerospace Research Centre, CZ-199 05 Praha, Czech Republic; § Center for Surface and Nanoanalytics (ZONA), Johannes Kepler University Linz, A-4040 Linz, Austria; ∥ University Service Centre for Transmission Electron Microscopy (USTEM), TU Wien, A-1040 Vienna, Austria; ⊥ Institute for Solid State Physics, TU Wien, A-1040 Vienna, Austria; # Applied Interface Physics, TU Wien, A-1040, Vienna, Austria

**Keywords:** Reduced Graphene nanosheets, l-Ascorbic acid reduction, De-Icing, Spray-coating, Corrosion Protection, Water uptake prevention, Aeronautical Application, Polymer filler

## Abstract

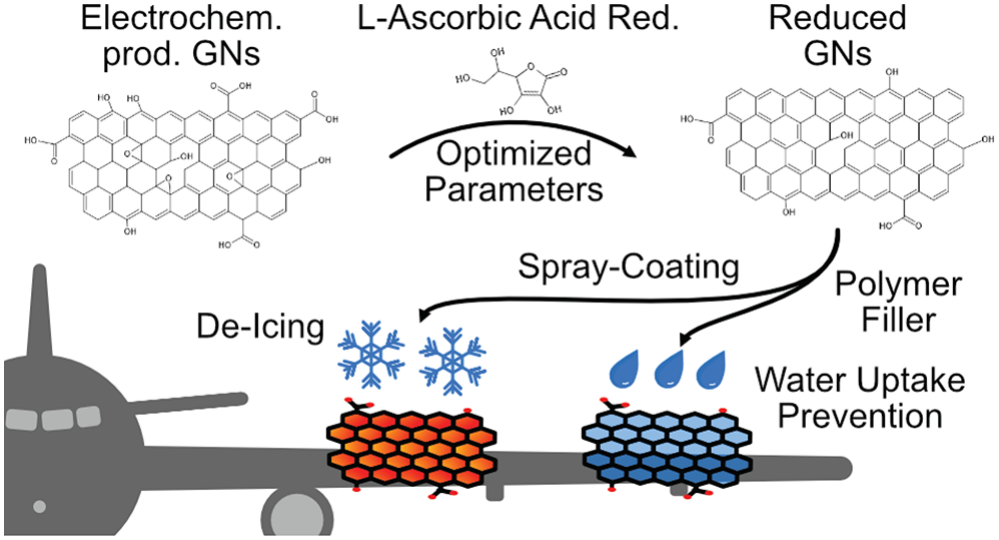

The aeronautical industry demands facile lightweight and low-cost solutions to address climate crisis challenges. Graphene can be a valid candidate to tackle these functionalities, although its upscalability remains difficult to achieve. Consequently, graphene-related materials (GRM) are gathering massive attention as top-down graphite exfoliation processes at the industrial scale are feasible and often employed. In this work, environmentally friendly produced partially oxidized graphene nanosheets (POGNs) reduced by green solvents such as l-Ascorbic Acid to rGNs are proposed to deliver functional coatings based on a glass fiber composite or coated Al2024 T3 for strategic R&D questions in the aeronautical industry, *i.e.*, low energy production, de-icing, and water uptake. In detail, energy efficiency in rGNs production is assessed via response-surface modeling of the powder conductivity, hence proposing an optimized reduction window. De-Icing functionality is verified by measuring the stable electrothermal property of an rGNs based composite over 24 h, and water uptake is elucidated by evaluating electrochemical and corrosion properties. Moreover, a mathematical model is proposed to depict the relation between the layers’ sheet resistance and applied rGNs mass per area, which extends the system to other graphene-related materials, conductive two-dimensional materials, and various substrates. To conclude, the proposed system based on rGNs and epoxy paves the way for future multifunctional coatings, able to enhance the resistance of surfaces, such as airplane wings, in a flight harsh environment.

## Introduction

In the current climate crisis, the aviation industry needs to provide solutions to lower its environmental impact according to the European Green Deal^1^ and the objectives expressed in the CORSIA (Carbon Offsetting and Reduction Scheme for International Aviation) program to reduce CO_2_ emissions.^2^ Few layers of two-dimensional materials (2DM), such as graphene, can exhibit outstanding mechanical and electrical functionalities, which make them ideal for low-cost and lightweight solutions.^3^ Consequently, in the past decade, graphene has found wide application in energy storage,^4^ wearable technologies,^5^ membrane technologies,^6,7^ and functional composites,^8^ just to mention some.

Upscaling production of single-layer flakes is still the main limitation to industrial application of graphene; hence, graphene-related materials (GRM) have been recently investigated.^9^ In the aviation industry, GRM have been tested to deliver De-Icing and Anti-icing,^10,11^ lightning strike protection,^12^ electromagnetic interference shielding,^13^ flame inhibition,^14^ corrosion protection,^15^ and mechanical improvement of composites.^16^

The goal of this work is to propose an innovative green pathway to address De-Icing and water-uptake prevention. Icing can cause major problems to an aircraft ultimately leading to a loss of control and fatal accidents. For instance, in 2009, the Air France Flight 477 crashed in the Atlantic Ocean because of ice formation in the pitot tube, which resulted in a wrong readout of the aircraft speed,^17^ or in 2017, the West Wind Aviation Flight 280 crashed due to ice nucleation points affecting lift and ultimately producing a loss of control.^18^ Many efforts have been made to implement De-Icing functionalities on composite aircraft;^19^ of those, thermoelectrical systems driven by 2DM appear to be the most suitable solution.^10,20−23^ The latter rely on Joule’s heating to increase the surface temperature above the freezing point and remove ice accretions. The technology is often implemented by integrating carbon materials (e.g. graphene-related materials, carbon nanotubes, graphite) into a polymer matrix.^22,23^ However, polymer implementation requires a significant amount of material usage, which favors the investigation of other low-material consumption methods such as spray-coating.^11,23^

The additional uptake of water within composites used in an aeronautical system may cause swelling, mechanical degradation, and corrosion of underlying structures.^24−26^ GRM based composites may prevent the diffusion of water due to their hydrophobic character.^25^ Thus, to exploit GRM for De-Icing and water uptake studies, it is necessary to clarify how to produce them and express specific functionalities. The most prominent graphene-related material is graphene oxide (GO), which is commonly produced by variations of Hummer’s methods for fast and large scale production.^27^ However, these methods employ nonenvironmentally friendly chemicals, which are not sustainable and detrimental to advancing a clean industrial production.^1^ An alternative way of exfoliating graphite is the electrochemical route using the intercalation of electrolyte compounds (e.g., sulfate ions, alkali ions) via an electrical field. This provides good upscale potential, avoids the use of toxic chemicals (e.g., manganese traces being considered harmful according to the literature^28^), and leads to less oxidation due to milder process parameters. Yet, it bears the disadvantage of providing a distribution of multiple layer numbers following exfoliation errors.^29−31^ Similar to GO, the presence of oxygen functional groups resulting from a prior exfoliation process enables material’s chemical modification with the trade-off of disturbing the aromatic backbone, which impedes both electrical and thermal conductivities of the material.

To overcome this problem, GO is reduced by chemical,^32−34^ electrochemical,^35^ thermal,^36^ solvothermal,^37^ plasma-assisted,^38^ and photoinduced^39^ methods in the literature, which are also applicable to the electrochemically derived material due to similar chemical properties of the functional groups. Chemical reduction often relies on reactants like hydrazine,^33^ hydroiodic acid,^40^ or NaBH_4_,^41^ which are dangerous for both the environment and human health.^42^ Consequently, green reactants are finding increasing application as they show comparable reduction potential to hydrazine.^32,43^ Among these, l-Ascorbic acid (AA) is the most promising one with high reduction potential, low material costs, and nontoxicity.

Understanding the role of reduction parameters (Temperature, Time, l-Ascorbic Acid concentration, pH) is critical for application of electrochemically exfoliated graphene nanosheets (GNs) at the industrial scale to minimize waste of energy and resources.

**Figure 1:**
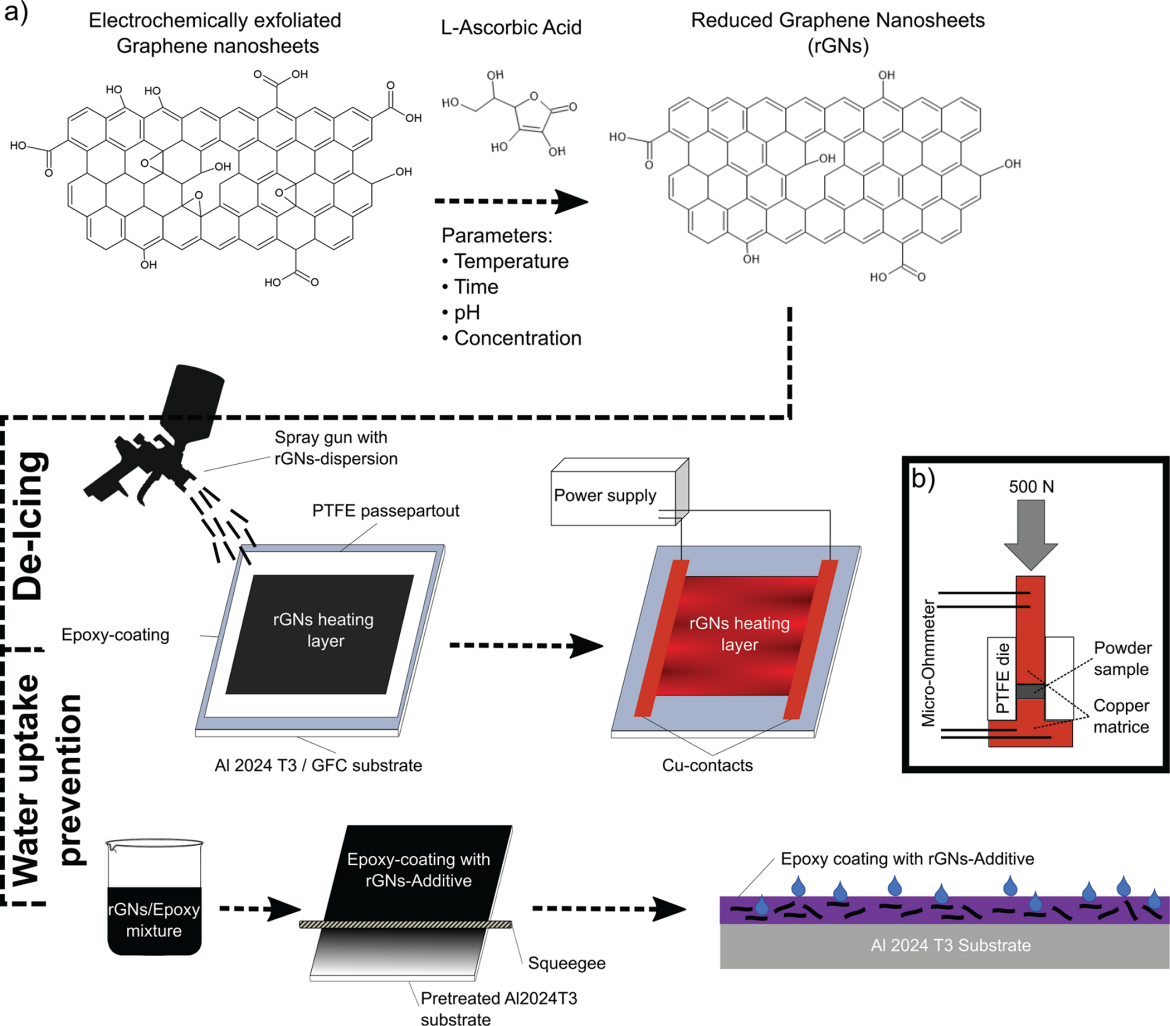
a) Scheme of work-flow: Reduction scheme of electrochemically exfoliated graphene nanosheets by l-Ascorbic acid to reduced graphene nanosheets (rGNs) as a function of reduction parameters Temperature *T*, time *t*, pH, and concentration of l-Ascorbic acid *c*_*AA*_. Subsequent use of rGNs in De-Icing application via spray-coating producing an rGNs heating layer and in Water uptake prevention via rGNs-additive in an epoxy coating; b) Setup for 4-point powder conductivity measurement including a copper matrice, a PTFE die, a micro-ohmmeter, and a force control system to apply 500 N.

In this work, we investigate AA reduction of our electrochemically produced partially oxidized graphene nanosheets (POGNs)^31^ and subsequent application of our environmentally friendly reduced graphene nanosheets (rGNs) to express De-Icing and water uptake prevention properties. Figure 1 a) shows the corresponding work-flow. First, we screen the AA reduction parameters and model the powder conductivity σ_*powder*_ providing an optimized reduction window with significant energy savings. Second, we analyze rGNs with multiple characterization methods to investigate the reduction mechanism. Third, we use the optimized rGNs to generate a conductive heating layer by spray-coating rGNs-dispersions on aeronautical relevant substrates and a layer of rGNs mixed into an aeronautical epoxy for water uptake prevention. The former is characterized in terms of De-Icing by cyclically measuring the electrothermal properties over 24 h, the latter for water uptake prevention by elucidating the water uptake, diffusion coefficient, and properties in a corrosive environment. Finally, we propose a model to predict the conductive properties of rGNs powders which we utilize to better understand the experimental findings and that could be possibly extended to other 2DM.

## Experimental Section

### Materials

All chemicals were used as purchased without further purification. Graphite rods (99% (metals basis)) and KBr (spectroscopy grade, ultrapure) were purchased at Alfa Aesar, NaOH (≥99%), l-Ascorbic acid (min 99%, p.a.), and *n*-butyl acetate (≥99%, for synthesis) were purchased at Carl Roth, H_2_SO_4_ (≥98%, Emsure) was purchased at Merck, Loctite EA 9390 epoxy resin was purchased at Dr.Losi, Al2024 T3 substrates were purchased at Robemetall, glass fiber composite substrates were purchased at Villinger R&D, and the NH_4_OH solution (25%) was purchased at VWR.

### Electrochemical Graphene Nanosheet Preparation

Electrochemical exfoliation of graphite took place as described in our previous work.^31^

### Reduction of POGNs by l-Ascorbic Acid

Graphene nanosheet reduction started by dispersing 1 g of electrochemically produced POGNs in 100 mL of deionized water via ultrasonication for 2 h. The dispersion was heated under stirring to the respective reaction temperature *T* followed by the addition of l-Ascorbic acid (2 or 5 g, equaling a concentration *c*_AA_ of 11.4 to 28.4 mM). The targeted pH according to the experimental plan was set with a 25% NH_4_OH solution. Stirring continued for the targeted reduction time *t*. Subsequent vacuum-filtration, washing with deionized water, and vacuum-drying at 50 °C and 10 mbar were used before further characterization.

To evaluate parameter influences, a fractional 2-factorial screening design (three repetitions, respective Experiments in Table S1 in the SI) was executed with the set of parameters summarized in Table 1. Subsequently, a Box-Behnken design on reduction using the parameters displayed in Table 2 was carried out (individual Experiments shown in Table S2 in the SI). Powder conductivity σ_*powder*_ was chosen as a response factor for optimization. An additional blank Experiment (Experiment 59) was executed at 95 °C for 15 min without l-Ascorbic acid.

**Table 1:** Parameters of Fractional 2-Factor l-Ascorbic Acid Reduction Screening Design

**Parameter**	**Lower Limit**	**Upper Limit**
Temperature *T* [°C]	25	95
l-Ascorbic acid concentration *c*_AA_ [mmol/L]	11.4	28.4
pH [1]	2.3	11.5
Time *t* [min]	15	120

**Table 2:** Parameters of Box-Behnken l-Ascorbic Acid Reduction Design

**Parameter**	**Lower Limit**	**Upper Limit**
Temperature *T* [°C]	55	95
Time *t* [min]	15	75
pH [1]	2.3	8.9

### Powder Conductivity Measurement

Figure 1 b) shows the setup used for powder conductivity measurements. Prior to each experiment, the copper base and piston were polished to remove the copper oxide layer, as well as the blank resistance checked. This was consistently kept below 50 *μΩ*, thus ruling out any influence on the measurement. With the Teflon matrice set on the copper base, 100 mg of powder sample was filled into the device. The copper piston was placed on the powder sample, and a Sauter FH 500 with a Sauter wheel test manual stand was used to apply 500 N force, resulting in a pressure of 4.42 MPa. Using a Micro-Ohmmeter (ndp technologies DRM-10A) for the 4-point method, the resistance *R* was measured. The pellet thickness *d*_Pellet_ was simultaneously evaluated by comparing the piston dissection to the blank measurement under pressure. The resistivity of each powder was measured three times to ensure reproducibility. Equation 1 was used to calculate the powder conductivity σ_*powder*_. *A*_Pellet_ describes the area of the pellet (= 1.13 cm^2^), and *R* describes the measured resistance with the Micro-Ohmmeter.
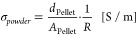
1

Similar measurement strategies for carbonaceous powder conductivity have been reported by Celzard et al.^44^ and Marinho et al.^45^

### Spray-Coating with rGNs

Figure 1 a) shows the setup for the spray-coating process. The as prepared rGNs were dispersed in *n*-butyl acetate at a concentration of 10 mg/mL under ultrasonication for 2 h. The substrate was placed on a metal plate preheated to 150 °C acting as a heat reservoir. To limit the coating area to 6 × 5 cm, a PTFE passepartout was used on top of the substrate. An IR lamp was placed above the sample to preserve the substrate temperature. A spray gun with pressurized air (3 bar) and a 1.3 mm nozzle were used to apply the dispersion onto the substrate. Spray intervals were chosen to ensure full solvent evaporation before applying the next layer. The powder layer was contacted with two copper adhesive tapes. To ensure mechanical stability, we used a wired K-bar (RK PrintCoat Instruments Ltd., UK) producing 100 m wet film thickness to apply a Loctite EA 9390 epoxy sealing on top of the powder layer. The sealing layer was cured at 93 °C for 220 min.

### Heating Tests

To test the samples’ thermoelectrical heating functionality, we used the setup depicted in Figure S1 in the SI. A power supply set the applied voltage. The heating process was monitored via an E5 Thermal imaging camera (FLIR systems, USA) together with a data logger. Furthermore, a Voltmeter and an Amperometer logged potential and current during experiments. For cycling experiments, the power supply was switched on and off every 10 min by a timing circuit (Siemens, Germany). The heating functionality was evaluated by connecting the sample to the power supply and measurement system followed by applying a defined potential (28 to 150 V depending on the sheet resistance) for defined periods of time (5–10 min) while monitoring the temperature change with the thermal imaging camera.

### Coating Al2024 T3 with rGNs-Modified Epoxy

rGNs were dispersed in *n*-butyl acetate at a concentration of 100 mg/mL under ultrasonication for 2 h. Then, Loctite EA 9390 epoxy part A was added while stirring and continuing ultrasonication for 2 h. The solvent was evaporated by vacuum-treatment at 40 °C and 80 mbar to reduce it to 10% content. Loctite EA 9390 epoxy part B was added in a ratio of 100:56 Part A:Part B. To achieve sufficient mixing and avoid entrapped air, vacuum-mixing was executed at 150 mbar for 30 min. Before the coating process, the Al2024 T3 substrate was degreased, etched, and pickled according to an industrial pretreatment protocol.^46^ The prepared rGNs/epoxy-mixture was applied with a wired K-bar (RK PrintCoat Instruments Ltd., UK) producing 150 m wet film thickness followed by curing at 93 °C for 220 min.

### Water Uptake Measurement of rGNs-Modified Epoxy Coatings

A Biologic SP-240 potentiostat (Biologic Sciences Instruments, France) in a three electrode cell configuration (Ag/AgCl in 3 M KCl reference electrode, platinum platelet counter electrode) served for recording electrochemical impedance spectroscopy (EIS). A quartz glass cylinder (diameter 4.7 cm) was fixed on the coated surface acting as an electrochemical cell and filled with a NaCl (3.5% w/w) solution as the electrolyte. EIS spectra were recorded after 0 h, 1 h, 2 h, 3 h, 4 h, 5 h, 6 h, 9 h, 12 h, and further every 6 h with 50 mV to 200 mV sinus amplitude at the open circuit potential (OCP) and a frequency range of 1 MHz to 10 mHz. In between EIS measurements, the OCP was measured. To determine water uptake, we used an equivalent circuit fitting with the usual circuit for failed coatings^47^ (see Figure S2 in the SI). The water uptake ϕ in *V*% was calculated using the Brasher-Kingsbury equation (see eq 2), where *C*_*t*_ describes the coating capacitance after time *t*, *C*_0_ describes the coating capacitance at the start, and ϵ_W_ describes the relative permittivity of water.^48,49^ The diffusion coefficient *D* of water through the coating is calculated by eq 3.^50^*k* describes the linear regression slope of the  diagram, *d*_coating_ describes the coating thickness, *C*_sat_ describes the coating capacitance at saturation, and *C*_0_ describes the coating capacitance at the start. The coating thickness *d*_coating_ was measured with a Dualscope FMP 40 (Fischer, Germany) using the eddy current method (DIN EN ISO 2360:2017)^51^ after calibration on an untreated Al2024 substrate. Figure S3 in the SI shows the corresponding coating thickness *d*_coating_ used for calculating the diffusion coefficient.
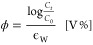
2
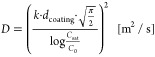
3

### Characterization

For further characterization of product powders and coatings, scanning electron microscopy (SEM), optical microscopy, transmission electron microscopy (TEM), electron energy loss near edge structure (ELNES), atomic force microscopy (AFM), Raman spectroscopy (Raman), X-ray photoelectron spectroscopy (XPS), powder X-ray diffraction (XRD), Infrared spectroscopy (IR), Zeta potential measurements, neutral salt spray test (NSS), and contact angle measurement were used.

To record SEM micrographs, a Sigma HD-VP (Zeiss, Germany) with a Everhart Thornley SE-electron Detector was utilized. Samples for cross-section analysis were prepared using standard metallographic methods (cutting, grinding, and polishing). The samples were sectioned with an IsoMet 4000 (Buehler GmbH, Switzerland), implemented to a self-curing acrylic potting resin SamplKwick FC, and polished through wet-rough and fine grinding processes using an AutoMet grinder-polisher (Buehler GmbH, Switzerland) with silicon carbide abrasive discs (granularity of P320–P600–P1200). Further polishing was performed with polishing cloths and a water-based monocrystalline diamond suspension (MetaDi) with particle sizes of 6 and 3 m, respectively, and final lapping with an aluminum oxide suspension (MasterPrep 0.05 m). Micrographs of the cross-section were recorded with a digital microscope VHX-6000 (Keyence, Japan). Atomic force microscopy was performed with an Asylum Research Cypher ES Atomic Force Microscope (Asylum Research, Oxford Instruments, Santa Barbara, CA) to acquire imaging of rGNs particles distributed onto a freshly cleaved mica surface. rGNs dispersed in *n*-butyl acetate by extensive ultrasonication was drop-casted onto the mica substrate heated between 125 and 130 °C in order to rapidly evaporate the solvent. Images were recorded in tapping mode using a silicon uncoated Tap300-G tip with force constant of 40 N/m. XPS measurements were executed with a Thetaprobe XPS system from Thermo Scientific (UK) to measure the oxygen content and ratio of oxygen containing groups in the POGNs starting material and the rGNs after optimization. To record TEM images and ELNES spectra, a TECNAI TF20 (FEI, Netherlands) equipped with a GATAN GIF Tridiem energy filter and spectrometer was used. The collection angle of the spectrometer was set to be 8.4 mrad. To measure ELNES spectra of both POGNs and rGNs, respectively, the TEM was operated in image mode. To record Raman spectra and determine the defect density of the produced powders, a LabRam Aramis from Horiba Jovin Yvon (Germany) with a 532 nm laser was utilized. XRD measurements were carried out on a PANanalytical Empyrean setup (Malvern Pananalytical, Germany) to determine the crystal structure, number of layers, and purity of the produced powder. The powders were prepared on an Si-Wafer and measured from 5 to 90°. The distribution of layer numbers *n* was determined as described in our previous work^31^ (fitting the (002) reflex and calculate *n* via Equations S1–S3 shown in the SI). The lateral crystallite size was calculated by fitting the (100) reflex and applying the Scherrer equation (see Equation S2 in the SI). IR-Spectra were measured on a Tensor 27 Hyperion (Bruker, USA) using the KBr-pellet method to determine the presence of functional groups. Zeta potential measurements of powder dispersions in water were executed using a Zetasizer Nano with disposable folded capillary cells (Malvern Pananalytical, Germany). The pH was set with 0.1 M HCl and 0.1 M NH_4_OH solutions. Roughness measurements of the substrate material were executed on a Perthometer S2 (Mahr GmbH, Germany) according to standard DIN EN ISO 12085:1998.^52^ A SaltEvent SC 1000 (Weisstechnik, Germany) was operated according to standard DIN EN ISO 9227:2017^53^ for the neutral salt spray test. Details on further sample preparation and analysis are described in the SI. The contact angle was measured with a Drop shape analysis system DSA10 Mk2 (Krss, Germany) with five drops of deionized water per sample. Furthermore, for experimental design and statistical analysis, the software Design expert 13 by Stat-Ease (USA) was employed. Monte Carlo Simulations to determine the increase of particles contact area during spray-coating were executed with Geogebra Classic 6 by GeoGebra GmbH (Austria; Detailed description in the SI).

## Results and Discussion

### Optimization of l-Ascorbic Acid Reduction

A fractional 2-factor screening design of the AA reduction helped to determine significant process parameters. This is displayed in Figure S4 a) of the SI by means of a Pareto chart. It shows that temperature *T*, time *t*, and pH as well as the interaction between *T* and *t* play a significant role during reduction. *T* is thereby the only positive linear factor increasing powder conductivity σ_*powder*_, as the AA concentration *c*_*AA*_ and other parameter interactions play only a minor role in the chosen parameter window. Hence, it is possible to reduce *c*_*AA*_ to a 2-fold excess by weight, saving a substantial amount of material compared to the maximal used 5-fold excess. The subsequent Box-Behnken Design established a quadratic model of the response surface area of σ_*powder*_ with the respective parameters shown in Table S3 in the SI.

**Figure 2:**
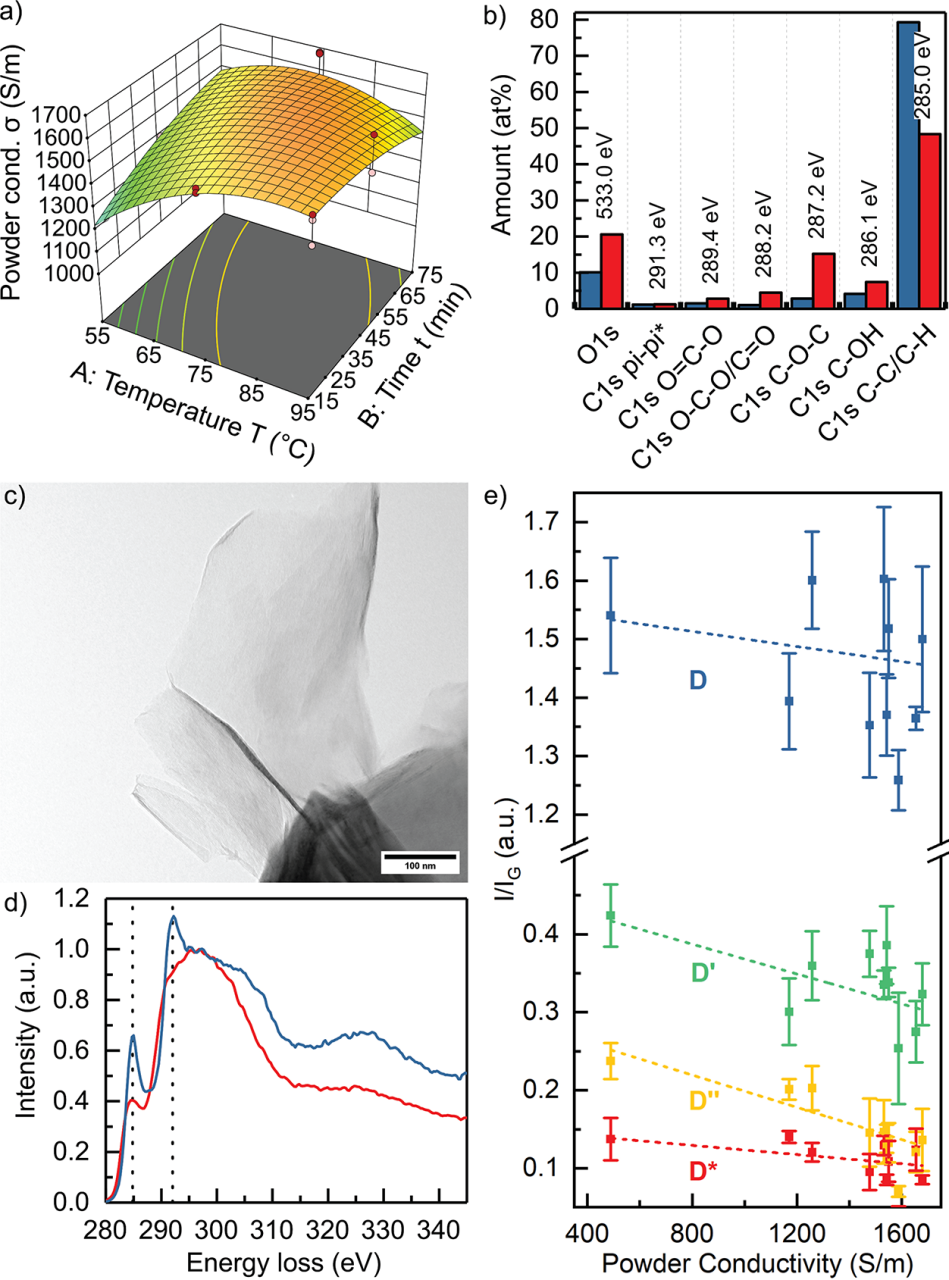
a) Resulting model of rGNs powder conductivity σ_*powder*_ against reduction temperature *T* and time *t* at pH 2.3; b) Deconvoluted XPS data showing the amount of functional groups in the electrochemically produced POGNs starting material (red) and the optimized rGNs (Experiment 60, blue); c) TEM micrograph of an rGNs flake; d) ELNES spectra of electrochemically produced POGNs starting material (red) and the optimized rGNs (Experiment 60, blue); e) *I*_D*_/*I*_*G*_ (red), *I*_*D*_/*I*_*G*_ (blue), *I*_*D*″_/*I*_*G*_ (yellow), and *I*_*D*′_/*I*_*G*_ (green) ratios against powder conductivity of POGNs starting material and rGNs samples.

Figure 2 a) depicts the response surface of σ_*powder*_ at pH 2.3 as a function of *T* and *t*. Similar graphs at pH 5.6 and pH 8.9 can be found in Figure S4 b-c) in the SI. For both *T* and *t*, negative quadratic coefficients are observed leading to the conductivity function’s negative curvature. Further, an interaction between *T* and *t* (labeled as AB in the Pareto chart, see Figure S4 a) in the SI) hampers σ_*powder*_ with an increasing reduction time at high reduction temperatures. This effect is related to additional agglomeration of particles. The described factors reveal an optimized reduction window with certain sets of reduction temperature and time (marked in orange in Figure 2 a)). The calculated optimum is at 81 °C and 50 min, resulting in σ_*powder*_ = 1599.0 S/m, at pH 2.3. Two confirmation Experiments (Experiments 60 and 61) using this set of parameters resulted in a mean measured σ̅_*powder*_ of 1592.6 ± 5.4 S/m, matching the prediction. Thus, reducing *T* from 95 to 81 °C results in about 20% lower energy consumption (from 9.59 kW/kg to 7.80 kW/kg; assuming 10% heat loss). In addition, using the resulting reduction window, it is possible to minorly trade-off conductivity for further energy savings by lowering the temperature and adjusting the time, respectively. Nevertheless, in this work, we selected the solution in the model that maximizes conductivity.

Furthermore, not only temperature and time but pH is found to play a role as well. The pH-related elements within the quadratic model appear with a negative linear slope and a positive quadratic factor. Initially, a pH-increase has an insignificant effect on the resulting σ_*powder*_. Nevertheless, at higher pH values, a minor increment in conductivity is observed because of the quadratic factor in the Box Behnken Model.

Our interpretation is that this effect is related to the dispersive nature of the POGNs starting material and the produced rGNs at different pH’s. Zeta potential measurements (see Figure S5 in the SI) of POGNs dispersions appear stable across the investigated pH range (2.3–8.9) with zeta potentials in the range of −33 to −44 mV. In our measurements, rGNs are stable until a pH of about 3 and start agglomerating at a lower pH as the zeta potential drops below −30 mV. Nonetheless, ammonia addition is avoided for further application due to the minor positive effect on conductivity as well as environmental and economical reasons.

A blank experiment (Experiment 59) without AA-addition validated the approach showing unchanged conductivity compared to the starting material.

The produced rGNs powders are characterized by a variety of techniques which are discussed in the upcoming paragraph.

POGNs and rGNs powders both show significant IR bands (see Figure S6 in the SI) at 3434 cm^–1^ (O–H stretching), 3000–2800 cm^–1^ (C–H stretching), 1723 cm^–1^ (C=O stretching), 1578 cm^–1^ (C=C stretching), 1385 cm^–1^ (C–O stretching of carboxylic group), 1214 cm^–1^ (C–O–C stretching), and 1124 cm^–1^ (C–OH stretching) in accordance with the literature.^31,54,55^ After reduction with AA, the intensities of O–H stretching, C–O–C stretching, and C–OH stretching are significantly lower indicating a successful process.

XPS spectra (Figure S7 in the SI) of POGNs and optimized rGNs allow quantification of the functional groups after fitting (Figure 2 b). Reaction with AA reduces the overall oxygen content from 20 at% to 10 at%. According to the literature, AA is assumed to attack primarily epoxy, carbonyl, and vicinal hydroxyl groups via an S_N_2-nucleophilic mechanism followed by an elimination reaction.^56^ This results in the predominant reduction of these in-plane functional groups as shown by XPS measurements. The reduction leads to restoration of the aromatic backbone and an increase in conductivity σ_*powder*_ by a factor of about 4. Functional groups, which are more likely located at the edge such as hydroxylic and carboxylic groups, are less likely attacked by AA and would offer the possibility of further chemical functionalization (e.g., activation followed by amidation^57^ or esterification^58^ of the carboxylic group).

A TEM micrograph shows the structure of a partly agglomerated rGNs flake in Figure 2 c). The flake appears wrinkled and partly folded with a size of about 250 nm. An SEM investigation of rGNs flakes dispersed and spray-coated on an Si wafer (see Figure S8 in the SI) shows an average flake diameter of about 433 ± 224 nm (averaged over 20 particles).

Figure 2 d) compares the C K-edge ELNES of POGNs starting material and rGNs. rGNs show two additional peaks at 284.8 and 292.0 eV corresponding to an increased number of sp^2^ carbon atoms present in the flakes, which are in agreement with the presented XPS results.

Determination of the crystal structure was executed via XRD measurements of the graphite used for exfoliation, the POGNs starting material, and different rGNs powders (see Figure S9 in the SI). As discussed in our previous work, electrochemical exfoliation of graphite to POGNs leads to asymmetric broadening of the (002) reflex at 26.57°. This indicates a distribution of different crystallite sizes in the *z*-direction accompanied by partial oxidation affecting the interlayer spacing. Contrary to GO, the reflex is not shifting to about 10° due to the milder exfoliation conditions. Only small parts of the POGNs material are significantly oxidized leading to a minor shakeup at 13.65°. Following the reduction process, only the asymmetric (002) reflex at 26.57° is observed. This reflex was fitted (see Figure S10 in the SI) according to previous works^31,59^ to calculate the amount of layer fractions as shown in Figure S11 in the SI. The distribution is similar to the one related to the electrochemical exfoliation process of the POGNs starting material showing no influence of the reduction on the crystallite thickness. The most dominant few-layered fraction shows a thickness of 2 nm corresponding to 4–5 layers. To determine the lateral crystallite size, the (100) reflex at 42.77° was fitted (see Figure S12 in the SI), and the crystallite size was calculated via the Scherrer equation (see eq S2 in the SI). Graphite particles used for the electrochemical exfoliation possess a lateral size of 31 nm. Due to the exfoliation and introduction of defects, this size is reduced to 22 nm in the POGNs material. Subsequent reduction does not influence the size significantly resulting in a mean lateral crystallite size of 24 nm of the optimized rGNs. This implies that the rGNs flakes with sizes of about 400 nm as observed in SEM and TEM are rather polycrystalline particles with sizes related to the graphitic material used for exfoliation.

Fragmentation of similar particles’ dispersions via ultrasonication is reported in the literature.^60^ Indeed, following extensive ultrasonic treatment of our produced rGNs, AFM measurements highlighted (see Figure S13 in the SI) a particle size distribution in line with the calculated crystallite sizes (22 nm wide, 2 nm height). Our interpretation of this result is that we observed full fragmentation of the particles along crystal boundaries down to the individual crystallites.

Raman spectroscopy yields information on the defect structures of POGNs and rGNs powders. Typical Raman spectra for the POGNs starting material and the rGNs product are shown in Figure S14 in the SI. By fitting the first order (1100–1800 cm^–1^) Raman region, intensities of the D*- (1150–1200 cm^–1^), D- (1350 cm^–1^), D′′- (1510 cm^–1^), G- (1576 cm^–1^), and D′-band (1615 cm^–1^) were determined. Figure 2 e) correlates the intensities of the defect-induced bands normalized to the G-band intensity with the powder conductivity σ_*powder*_. Different reduction parameters only minimally influence D-band intensity compared to POGNs. Furthermore, they show almost no correlation to powder conductivity. Our interpretation is that the D-band reflects the A_1g_ breathing mode correlating to defects of the basal graphene plane like destroyed carbon hexagons.^61,62^ These carbon-lattice defects are related to the top-down electrochemical exfoliation process to produce POGNs, which results in a distribution of various layer numbers, in-plane defects, and oxidation of graphite starting material, as described in the literature.^31,63^ Although the reduction with AA reduces in-plane defects in the form of functional groups, carbon lattice disorder remains. Therefore, reduction slightly influences the intensity of the D-band and loosely correlates to σ_*powder*_. The D*-band is related to sp^2^-sp^3^ bonds at the edges.^61^ As shown in Figure 2 e), the D*-band shows little correlation to powder conductivity σ_*powder*_, because the reduction is more selective to in-plane functional groups than to edge functional groups. This is in accordance with XPS measurement (Figure 2 b)), suggesting a higher decrease of in-plane oxygen functionalities. Regarding the defect-related interbands D′′ and D′, the respective intensities show good correlation with σ_*powder*_. The D′′-band originates from amorphous lattices. Its intensity is therefore inversely proportional to crystallinity.^61^ The D′-band has been correlated to crystal defects resulting from rings with different C numbers and C–O bonds.^61^ AA reduction decreases the number of crystal defects and C–O bonds restoring aromaticity and subsequently increases σ_*powder*_. Therefore, Raman spectroscopy appears a viable option for in-line quality control in industrial rGNs production by AA.

### rGNs-Based Electrothermal De-Icing Layer

**Figure 3:**
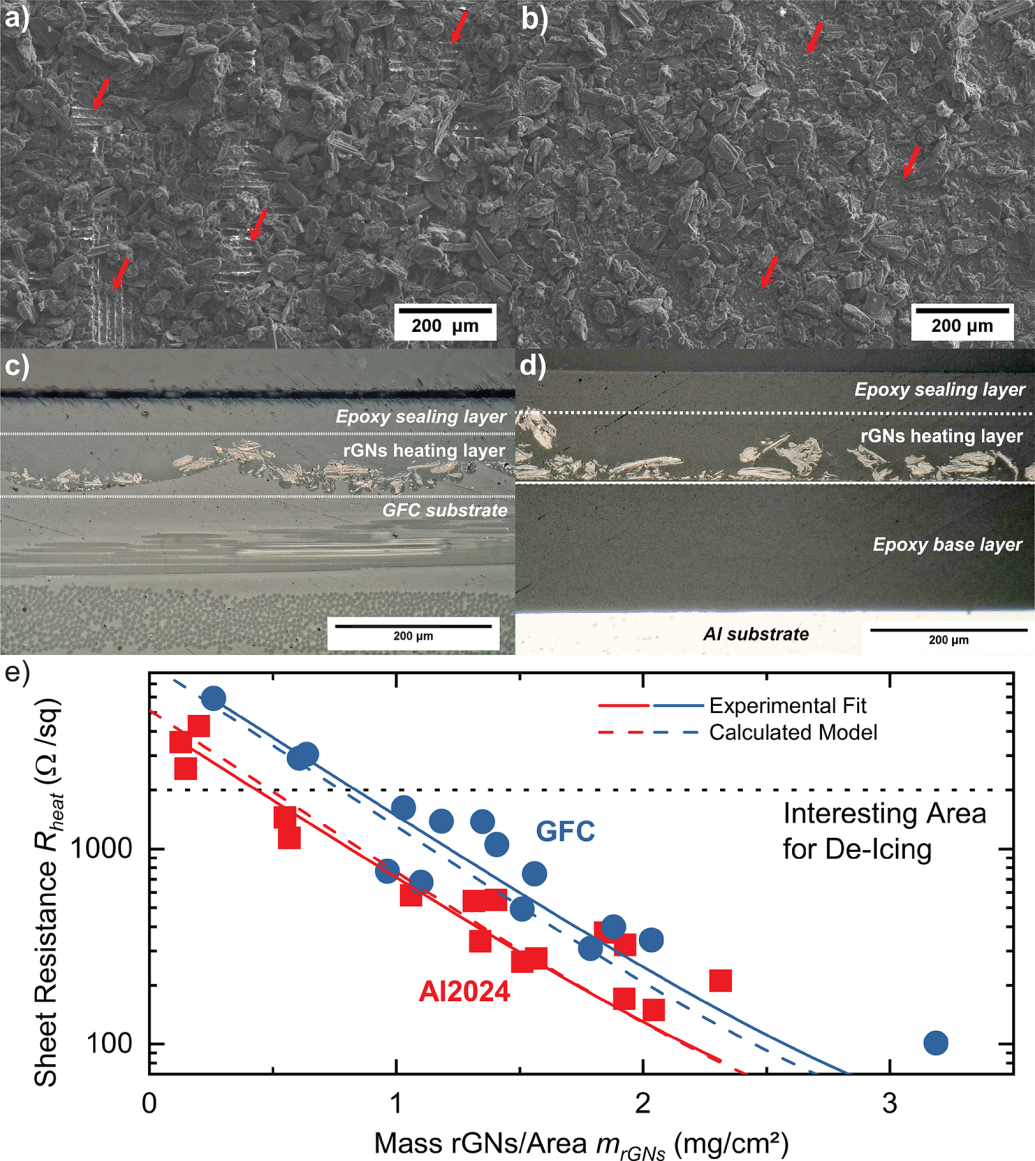
a-b) SEM micrograph of a spray-coated rGNs powder layer on a) a glass fiber composite substrate, b) an epoxy-coated Al2024 substrate; c-d) an LOM micrograph of De-Icing panels cross-cut with c) a glass fiber composite substrate, rGNs heating layer, and an epoxy sealing, d) an epoxy-coated Al2024 substrate, rGNs heating layer, and an epoxy sealing; e) Sheet resistance *R*_*heat*_ of De-Icing panels produced by rGNs spray-coating on a glass fiber composite (blue) and an epoxy-coated Al2024 (red) substrate against the applied rGNs mass per area *m*_*rGNs*_; solid lines describe the exponential fit of experimental data, and dashed lines describe the calculated model according to eq 9.

Based on the optimized rGNs powder, a thermoelectrical De-Icing device is produced via Spray-coating. Figure 3 a-b) shows the SEM micrographs of spray-coated layers on glass fiber composite (GFC) and epoxy-coated Al2024 substrates, respectively. The rGNs flakes are distributed on the surface in random orientation. Minor inhomogeneities (marked with red arrows) within the layer are attributed to the manual spray-coating process. Immediate evaporation of the solvent is ensured by preheating the substrate about 20 °C above the solvent’s evaporation point to avoid the coffee-ring effect.^64^ To sustain this temperature, a heat reservoir below the substrate, an infrared lamp, or a heat gun was used.

Figure 3 c-d) depicts cross-cuts of epoxy-sealed powder layers on GFC and epoxy-coated Al2024 substrates. The GFC substrate (Figure 3 c) shows two layers of glass fiber fabric containing an epoxy layer about 50 m thick above the fabrics. It exhibits a significantly higher surface roughness (R_a_ = 5.87 ± 1.29 m) than the almost completely flat epoxy-coating on Al2024 (R_a_ = 0.02 ± 0.01 m; see Table S5 in the SI). The spray-coated layer’s thickness *t*_*layer*,*GFC*_ is 28 ± 3 m with rGNs powder flakes randomly distributed on the GFC surface forming a continuous layer. The epoxy sealing layer covers the particles to achieve mechanical stability. Figure 3 d) depicts the cross-cut of a De-Icing panel prepared on an epoxy-coated Al2024 substrate. The rGNs layer shows a thickness *t*_*layer*, *Al*2024_ of 29 ± 4 m and is situated between an epoxy base and a sealing layer. Similar to the GFC substrate, the particles are randomly distributed forming a continuous, conductive layer on the epoxy coating.

Summarizing multiple test panels, Figure 3 e) depicts the sheet resistance of the powder layer *R*_*heat*_ against the applied mass of rGNs per area *m*_*rGNs*_. Thereby, an effective electrothermal heating layer requires a resistance of 5–2000 Ω/sq. By applying at least 0.5 mg/cm^2^ rGNs powder on an epoxy-coated Al2024 or at least 0.9 mg/cm^2^ on a GFC substrate, we are able to measure the set resistance.

To further model the correlation between *R*_*heat*_ and *m*_*rGNs*_, the measured data are fitted with an exponential function (see eq 4) in three parameters *A*, *B*, and *C* showing R^2^ ≥ 0.90 (see Table 3).
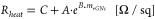
4

**Table 3:** Parameters *A*, *B*, and *C* of the Exponential Relation (*y* = *C* + *A* · *e*^*B*·*x*^) between Sheet Resistance *R*_*heat*_ and Mass rGNs per Area *m*_*rGNs*_ for De-Icing Panels with Epoxy-Coated Al2024 and GFC Substrates and Comparison of Fit According to Experimental Data and Calculation from Powder/Substrate Characterization^a^

**Substrate**	**Epoxy-coated Al2024**		**GFC**	
	**Fit**	**Calculated**	**Fit**	**Calculated**
*A*	4445	4256	9426	7380
*B*	–1.860	–1.822	–1.865	–1.822
*C*	22.42		22.42	
R^2^ (COD)	0.90	0.90	0.95	0.87

a R^2^ of experimental fit and calculated model to experimental data.

*A* describes the amplitude of the exponential model as defined in eq 5. *A* includes powder-dependent information such as the powder conductivity σ_*powder*_, the lateral crystallite area *A*_*Crystallite*_, the crystallite thickness *t*_*Crystallite*_, and the Krenchel orientation factor η_0_ for randomly distributed 2D materials.^65^ The denominator in eq 5 describes the nature of the established particle network due to σ_*powder*_ related to the bulk conductivity, *A*_*Crystallite*_ describes the conductive path per crystallite, and *t*_*Crystallite*_ describes the nonconductive direction within the crystallite. Hence, *A*_*Crystallite*_/*t*_*Crystallite*_ is associated with the anisotropic conductive behavior of graphene-related materials shown in the literature.^45^

Furthermore, substrate-dependency is expressed by a roughness-related factor *r* (see eq 6), where *R*_*z*_ is the average maximum peak–valley height of the profile, and *n*_*Peaks*_ is the number of peaks extrapolated from the measurement length (here 0.8 mm) to the layer width. *d*_*electrodes*_ is the distance between the heating layer’s electrical contacts to the power supply (≡ powder layer width). Hence, *r* is associated with the increased distance between the electrical contacts induced by the substrate surface roughness. This results in an increased amount of particles necessary to establish electrical connection. For example, rough GFC substrates reveal a roughness factor *r* of 1.73 compared to the nearly ideally flat epoxy-coating (see Table S5 in the SI).
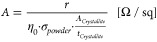
5
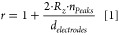
6

The exponent *B* in eq 4 depends on rGNs-powder characteristics according to eq 7, where *ΔA*_*cont*/*Crystallite*_ is the increase of contact area per crystallite, *V*_*Crystallite*_ is the crystallite volume, and ρ_*powder*_ is the bulk density of rGNs. It describes the effective increase in the contact area between the rGNs particles and therefore relates to the number of conductive pathways through the layer. *ΔA*_*cont*/*Crystallite*_ is thereby simulated via Monte Carlo Simulations (described in detail in the SI), while the applied number of crystallites per gram is calculated via the crystallite volume *V*_*Crystallite*_ and the bulk density of rGNs ρ_*powder*_.
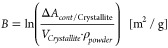
7

*C* in eq 4 describes the constant factor of the exponential function (see eq 8), where σ_*powder*_ is the powder conductivity, and *t*_*layer*_ is the thickness of the heating layer. It corresponds to the minimal sheet resistance *R*_*heat*_, which one can reach at *t*_*layer*_ and σ_*powder*_. Considering a powder conductivity of 1593 S/m (from powder conductivity measurements) and a layer thickness of about 28 m (Optical microscopy cross-cut analysis), *C* was set to 22.42 Ω/sq.
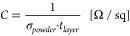
8

Overall, the sheet resistance of the heating layer *R*_*heat*_ can be related to the applied mass of rGNs per area *m*_*rGNs*_ through eq 9.

We applied the model to verify the conductive properties of our rGNs-layer by employing it in the fit experimental data or educated guesses if needed. Table S6 in the SI summarizes the parameters utilized in the model to fit the experimental data of *R*_*heat*_ against *m*_*rGNs*_ of the thermoelectric de-icing layer coated on epoxy-coated Al2024 and GFC (see Table 3). Figure 3 e) shows a good agreement between the model and the experimental data.
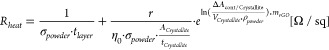
9

Consequently, the proposed model allows prediction of the feasibility of heating layers with different grades of rGNs in terms of lateral size, thickness, and conductivity, as well as different substrates, which is crucial for industrial application. More generally, the presented model may potentially predict powder properties of other 2DM as well, with the added value of utilizing data obtained from rather simple characterization methods such as powder conductivity, roughness, and XRD measurements. It is assumed that within the polycrystalline flakes, the size of the individual crystallites is a key factor in determining the conductive network. Considering possible harmful effects of few-μm sized flakes,^66^ the use of sub-μm material is favorable within polycrystalline systems.

Optimization is therefore driven by alternating graphite starting material toward a bigger lateral crystallite size at a similar flake size. In our system, a concentration of about 1.5–2.5 mg/cm^2^ is assumed to be a suitable range, considering economic reasons and weight savings.

Therefore, by applying 2 mg/cm^2^ and a typical over spray of 30% in industrial processes, rGNs material costs are at about 16 €/m^2^ as a result of the low-cost electrochemical production of POGNs^31^ and optimized reduction with AA. The detailed energy and cost calculation in Tables S7 and S8 are shown in the SI. Up-scale to industrial production potentially reduces costs further. This shows the economical feasibility of the system and also extends the possible range of application to other sectors (e.g., interior heating, automotive applications).

**Figure 4:**
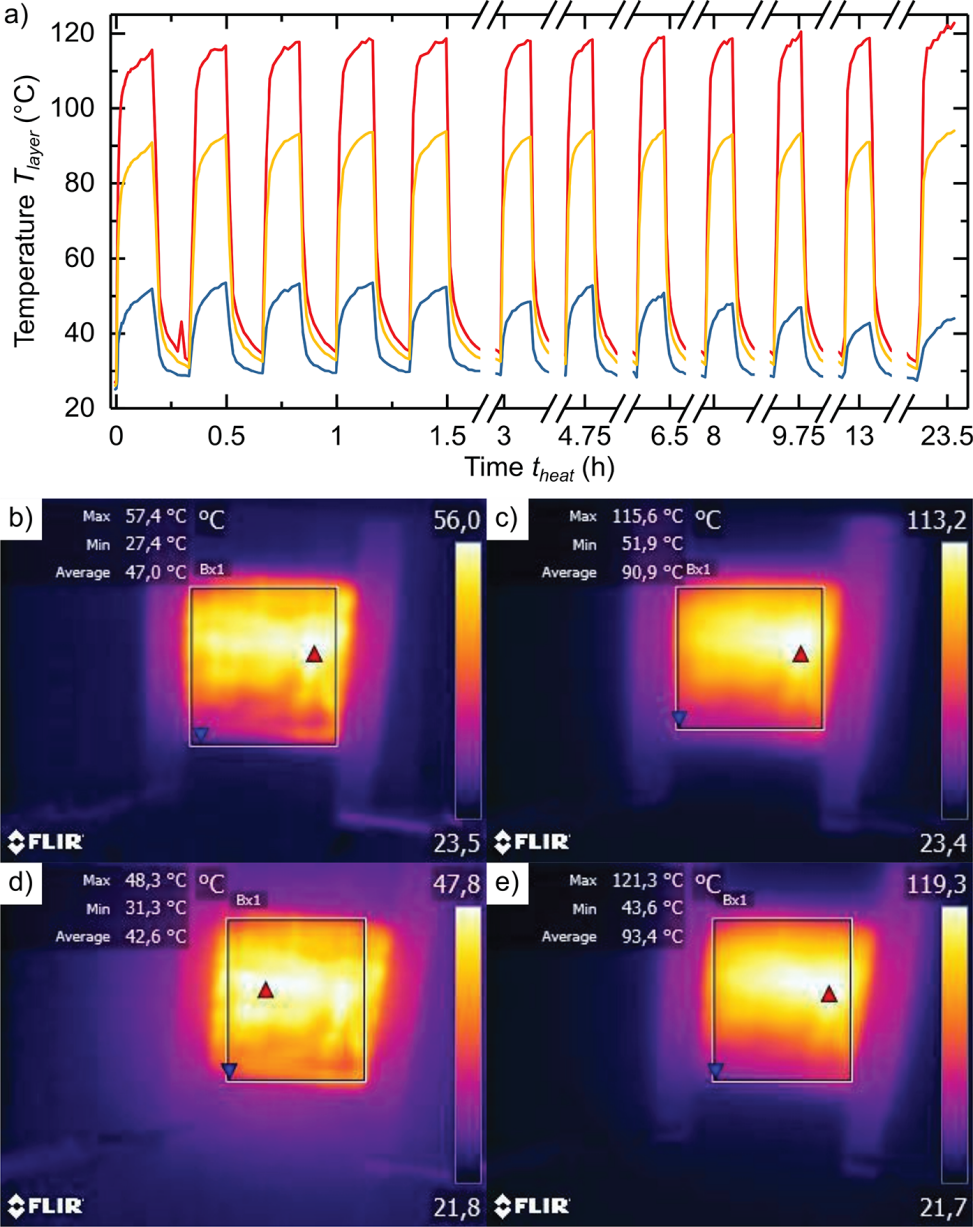
a) Temperature profile of the heating layer during the cyclic heating test with maximum (red), average (yellow), and minimum (blue) temperature against time; b-c) De-Icing Panel thermal image after 15 s (b) and 10 min (c) heating times during the 1st cycle; d-e) De-Icing Panel thermal image after 15 s (d) and 10 min (e) heating times during the 71st cycle.

To test the De-Icing functionality, we carried out thermoelectrical heating tests, as shown in Figure 4, based on a heating layer with 1.56 mg/cm^2^ rGNs applied on a GFC substrate. A potential of 100 V was applied to the layer resulting in a fast Joule’s heating process. The temperature profile (Figure 4 a)) was recorded with an IR camera showing the maximum (red), average (yellow), and minimum temperature (blue) of the heating layer. The temperature rises with an initial heating rate of about 23.5 °C/min during the first 2 min reaching about 80 °C. Further heating after this initial stage for up to 10 min resulted in a moderate temperature increase to 91 °C at a heat rate of 1.17 °C/min. The temperature of the hottest spot at the end of the heating cycle is 116 °C indicating uniform and consistent temperature distribution. After the 10 min heating cycle, we carried out a cooling cycle for the same time by deactivating the power supply. Heating and cooling were repeated 70 times to investigate repeatability of the electrothermal functionality. Figure S16 in the SI shows the detailed temperature profile and intermediate thermal images at the end of cycles 1, 10, 20, 30, 40, and 71.

Figure 4 d) shows the thermal image after 15 s of the 71st heating cycle. The heat distribution appears unchanged compared to the first cycle with the corresponding temperatures and heat rates (25.0 °C/min in the first 2 min and 1.25 °C/min further) being within usual measurements’ deviation. After applying the current for 10 min, the thermal image (Figure 4 e)) still resembles the other images showing stable heat distribution with no sign of degradation. In the subsequent cycles, the temperature slightly increases as the substrate does not reach room temperature during the cooling cycle, leading to an increment in the peak temperature over time. Overall, the proposed system based on inexpensive materials shows reliable heating functionality during multiple cycles.

Ice pellets are frozen on top of the test panels (example shown in Figure S17 in the SI), to confirm operational work below the freezing point. Similar to prior nonfreezing conditions, an initial rapid heat-up of the layer is observed. Thereby, surface temperature crossing the freezing point and subsequent loss of contact of the ice platelet - meaning successful De-Icing - varied between 30 and 200 s depending on the substrate and applied power (60 s in this example). This indicates temperature-independent heating functionality suitable for in-flight temperatures down to −40 °C.

To further characterize the De-Icing systems, we calculated the necessary electrical energy per area *E*_*el*_/*A* to achieve a certain temperature increase *ΔT*_*layer*_. Figure S18 in the SI represents *E*_*el*_/*A* against *ΔT*_*layer*_ during multiple heating tests on panels comprising epoxy-coated Al2024 (red) and a GFC substrate (blue), respectively. Due to the higher heat capacity of the material, epoxy-coated Al2024 consumes 2.21 J/K·cm^2^, whereas GFC uses 0.93 J/K·cm^2^, which highlights the role of the underlying structures for successful De-Icing systems.

### rGNs-Based Epoxy Coating for Preventing Water Uptake

**Figure 5:**
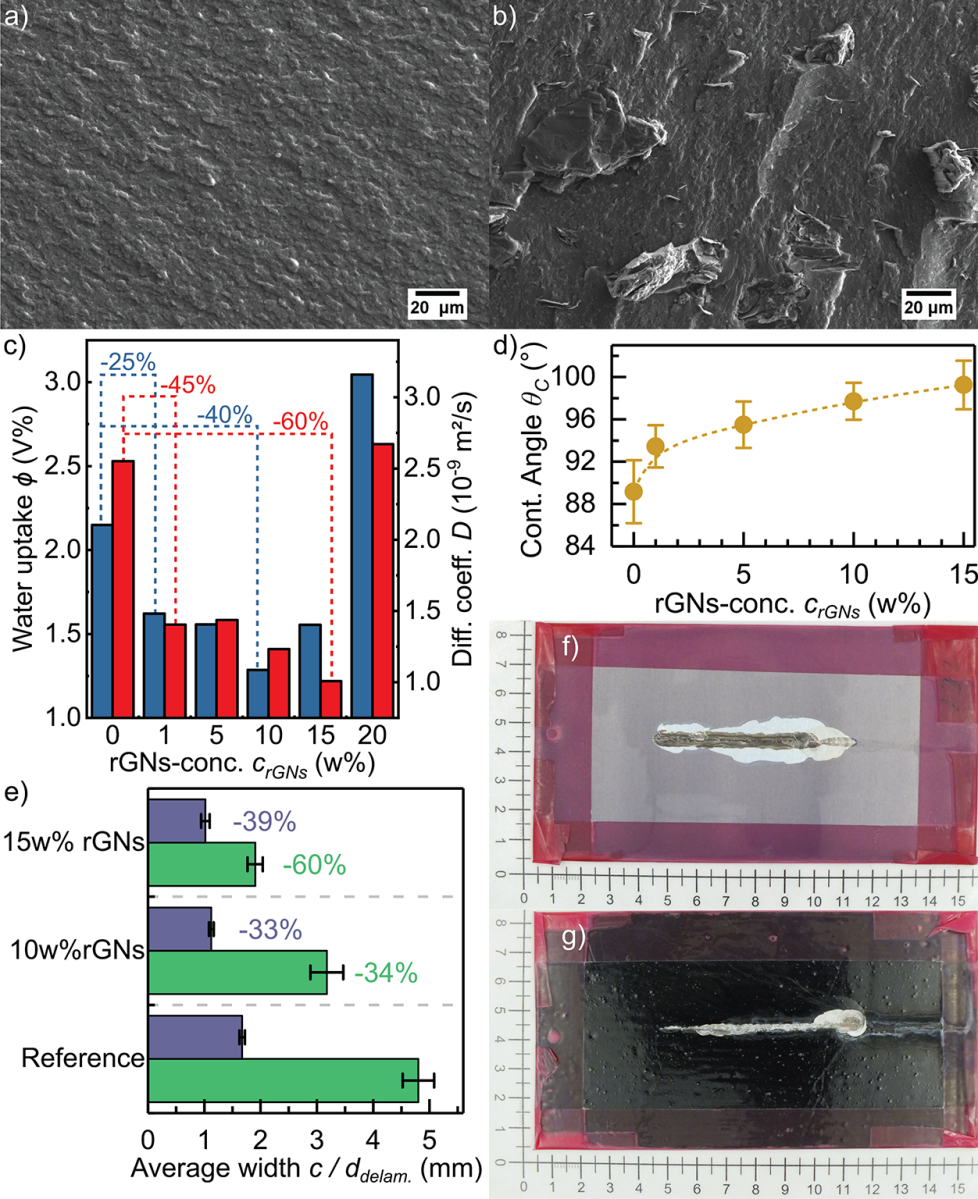
a) SEM micrograph of reference epoxy-coating break surface; b) SEM micrograph of the break surface of epoxy-coating with 15 w% rGNs-addition; c) water uptake ϕ (blue) and diffusion coefficient *D* of water (red) against the concentration of rGNs-additive *c*_*rGNs*_; d) Contact angle θ_*c*_ of deionized water on epoxy-coatings against the rGNs additive concentration *c*_*rGNs*_ in the coating; e) corrosion degree *c* (violet) and mean width of delamination *d*_*delam*._ (green) at the scribe after 2026 h of a neutral salt spray test for the reference epoxy coating and coating with 10 w% and 15 w% rGNs-addition; f-g) Neutral salt spray test reference sample (f) and sample with 15 w% rGNs-additive (g) after a 2026-h exposure and removal of the delaminated coating material.

Figure 5 a) shows a SEM micrograph of the epoxy reference coating’s cross-section after preparation by cryobreak. The break surface appears smooth with small breakouts distributed across it. The cross-section of the coating containing 15 w% rGNs appears instead more irregular. The break surface presents more of a flake-like topography, mimicking the morphology of rGNs. The additive is thereby uniformly and randomly distributed within the modified epoxy coating structure.

To investigate water uptake and diffusion properties of rGNs-modified epoxy-coatings, we compared increasing rGNs-additive concentrations *c*_*rGNs*_ (1; 5; 10; 15; 20 w%) to a pure epoxy reference coating. Figure 5 c) depicts the nonlinear water uptake trend of the coatings as a function of *c*_*rGNs*_. Figure S19 in the SI shows the detailed progression of the water uptake ϕ against time *t* for each sample, showing a significant decrease from 2.15 V% to 1.62 V% (i.e., by almost 25%) when adding 1 w% rGNs. By increasing the rGNs concentration, ϕ reduces to 1.29 V% at 10 w% rGNs, which is 40.0% less compared to the reference sample. We conclude that this effect is related to the presence of rGNs and their ability to increase hydrophobicity of coatings. Our hypothesis is further confirmed by contact angle measurements shown in Figure 5 d), where a steady increase in the contact angle can be appreciated as a function of rGNs-concentration. We observed that this trend is not linear. By adding more than 10 w% of rGNs, an increasing water uptake could be recorded due to the growing number of defects in the coating. Our interpretation is that the 2D platelets act as an impermeable barrier for water and extend diffusion paths through the coating. This effect is in accordance to other graphene-based coatings described in the literature.^67,68^

Indeed, the diffusion coefficient *D* shows a similar nonlinear trend: when adding 1 w% rGNs, diffusion already decreases by 45% compared to the reference. This decrease continues until 15 w% rGNs reaching 60.4%. Adding further rGNs results in defects within the coating structure, which translate in an increased diffusion coefficient.

To evaluate the corrosion resistance of rGNs-modified coatings, we executed a neutral salt spray test^53^ on coatings comprising 10 w% and 15 w% rGNs-additive as well as a reference coating. Figure 5 e) shows the average width of delamination *d*_*delam*._ (green) and degree of corrosion *c* (violet) for these samples according to standard DIN EN ISO 4628-8:2012 (described in the SI). Figure 5f-g) depicts the corresponding images of the samples after a 2026-h exposure and removal of the delaminated coating. Images of the samples (Reference, 10 w%, 15 w% rGNs) at different exposure times are presented in Figures S20–S22 in the SI. No blistering, cracking, flaking, or filiform corrosion is visible at the macroscale on the coated area aside from the scribe. Increasing corrosion and delamination occur at the scribe with ongoing exposure. Both the mean width of delamination and corrosion degree are significantly reduced when adding rGNs. The effect is larger at 15 w% compared to 10 w%. In total, adding 15 w% rGNs to the epoxy coating reduces the corrosion degree by 39.1% and the mean width of delamination by 60.4%. Clearly, the increased resistance to the corrosive environment correlates to the decreased water uptake and diffusion coefficient, proving the feasibility of the proposed rGNs for advanced coatings. With the low cost production route, concentrations of 10–15 w% would add additional costs of about 8–12 €/m^2^ to the coating system (150 g/m^2^). Considering already a significant effect on water uptake prevention at 1 w% rGNs-addition, the used concentration in the industry will be determined by the functional requirements and economic factors.

## Conclusions

The present work investigates the parameters influencing POGNs reduction by l-Ascorbic acid (AA) and proposes a green, cheap, electrothermal, and corrosion protecting reduced graphene nanosheets (rGNs) coating for application in the aeronautical industry.

By applying a Box-Behnken-model for the AA reduction, we could define an optimal reduction window, which can save costs for energy and chemicals. AA treatment shows good reduction of in-plane functional groups, lower defect density, and enhanced powder conductivity σ_*powder*_ without affecting the distribution of layer numbers. We discussed Raman spectroscopy and powder conductivity measurements as viable options for quality management on an industrial scale with in-line scanning and a simple test procedure.

We demonstrated a facile spray coating application to produce De-Icing coating. We proposed a mathematical model for the experimental data to elucidate the relation between sheet resistance of the heating layer *R*_*heat*_ and the applied mass of rGNs per area *m*_*rGNs*_, which could predict further GRMs and 2DM behaviors.

The system shows reliable heating functionality over multiple cycles and at temperatures below 0 °C. Due to the optimized production and the use of only 2 mg/cm^2^, rGNs material costs are lower than 16 €/m^2^.

We proved a reduction of water uptake by 40% for an rGNs/epoxy-system and further corrosion resistance. The latter lowered the corrosion degree by 39.1% and the recorded mean width of delamination by 60.4% in salt spray chamber tests.

In conclusion, we proved multiple functionalities of a coating based on green produced rGNs with exceptional low material cost (16 €/m^2^), electrothermal ability for de-icing applications, and intrinsic hydrophobicity for water uptake prevention. Moreover, we proposed a general model to predict the conductive behavior of our produced spray-coated rGNs layer, which can be extended to other families of 2DM and become a valuable tool for a fast and facile 2DM selection in research and development.

As an outlook, the subsequent steps will consist in merging the properties found in single layered structures for a true multifunctionality, which possibly could include lightning strike and fire protecting functionalities to deliver an advanced coating for the aeronautical industry.

## Supporting Information Available

Experimental: Individual Experiments of Experimental Designs, Heating Test Setup, Equivalent Circuit for EIS, Coating thickness, Description of XRD fitting, Neutral salt spray test. Results: l-Ascorbic Acid Reduction (Pareto-Chart, Box-Behnken Design, Zeta Potential measurement, SEM, IR, XPS, XRD, AFM, Raman), De-Icing (Monte Carlo Simulation, Roughness and fitting data of De-Icing panels, Cost calculation, Cyclic Heating Test, Freezing Test, Energy per Temperature change), Water Uptake and Corrosion Test (Water Uptake against time, NSS Test panels) (PDF)
