# Supplementary material for: l-Ascorbic Acid Treatment of Electrochemical Graphene Nanosheets: Reduction Optimization and Application for De-Icing, Water Uptake Prevention, and Corrosion Resistance
Source: ACS Appl Mater Interfaces. 2023 Apr 26;15(18):22471–84. doi: 10.1021/acsami.2c22854 (PMC10176320; doi:10.1021/acsami.2c22854)
Supplement: Supplementary file 1 — am2c22854_si_002.pdf [file am2c22854_si_002.pdf]

Supporting Information: L-Ascorbic Acid treatment of Electrochemical Graphene Nanosheets: Reduction Optimisation and Application for De-Icing, Water Uptake Prevention and Corrosion Resistance

Markus Ostermann,[*,†] Pierluigi Bilotto,[*,†] Martin Kadlec,[‡] Jürgen Schodl,[†] Jiri Duchoslav,[†,¶] Michael Stöger-Pollach,[§,‖] Peter Lieberzeit,[⊥] and Markus Valtiner[†,#]

†*CEST GmbH, Centre for Electrochemical Surface Technology, A-2700, Wiener Neustadt, Austria*

‡*VZLU – Czech Aerospace research centre, CZ-199 05, Praha, Czech Republic*

¶*Center for Surface and Nanoanalytics (ZONA), Johannes Kepler University Linz, A-4040 Linz, Austria*

§*University Service Centre for Transmission Electron Microscopy (USTEM), TU Wien, A-1040 Vienna, Austria*

‖*Institute for Solid State Physics, TU Wien, A-1040 Vienna, Austria*

⊥*Institute of Physical Chemistry, University of Vienna, A-1090, Vienna, Austria*

#*Applied Interface Physics, TU Wien, A-1040, Vienna, Austria*

E-mail: markus.ostermann@cest.at; pierluigi.bilotto@cest.at

Experimental

Reduction of partially oxidized graphene nanosheets by L-Ascorbic acid

Table S1: Individual Experiments of the preliminary fractional 2-factor screening design of L-Ascorbic acid reduction

Experiment	Temperature T [°C]	concentration (Ascorbic acid) c_{AA} [mmol/l]	pH [1]	Time t [min]	Powder conductivity σ_{powder} [S/m]
POGNs Starting material	-	-	-	-	489
1	25	28.4	2.3	120	1120
2	95	28.4	11.5	120	825
3	95	11.4	2.3	120	1053
4	95	28.4	2.3	15	1570
5	95	11.4	11.5	15	1469
6	25	11.4	2.3	15	1123
7	25	28.4	11.5	15	972
8	25	11.4	11.5	120	922
9	95	11.4	11.5	15	1324
10	95	28.4	2.3	15	1549
11	25	11.4	11.5	120	974
12	25	28.4	11.5	15	955
13	95	11.4	2.3	120	1008
14	95	28.4	11.5	120	721
15	25	28.4	2.3	120	846
16	25	11.4	2.3	15	708
17	95	11.4	2.3	120	1170
18	25	28.4	11.5	15	991
19	25	11.4	2.3	15	1011
20	95	28.4	11.5	120	848
21	95	28.4	2.3	15	1445
22	25	11.4	11.5	120	898
23	25	28.4	2.3	120	962
24	95	11.4	11.5	15	1038

Table S2: Individual Experiments of the Box-Behnken design of L-Ascorbic acid reduction

Experiment	Temperature T [°C]	Time t [min]	pH [1]	Powder conductivity σ_{powder} [S/m]
25	55	45	8.9	1320
26	95	75	5.6	1330
27	75	15	2.3	1532
28	95	45	2.3	1495
29	95	45	8.9	1543
30	75	45	5.6	1413
31	55	75	5.6	1340
32	75	75	2.3	1679
33	75	45	5.6	1387
34	75	45	5.6	1438
35	75	15	8.9	1478
36	55	45	2.3	1390
37	55	15	5.6	1094
38	75	45	5.6	1338
39	95	15	5.6	1363
40	75	45	5.6	1365
41	75	75	8.9	1248
42	55	75	5.6	1316
43	75	75	2.3	1674
44	55	45	8.9	1258
45	95	75	5.6	1186
46	75	75	8.9	1470
47	55	45	2.3	1326
48	75	45	5.6	1390
49	95	45	8.9	1410
50	95	15	5.6	1348
51	55	15	5.6	1122
52	75	45	5.6	1436
53	75	45	5.6	1471
54	95	45	2.3	1654
55	75	15	8.9	1343
56	75	15	2.3	1513
57	75	45	5.6	1379
58	75	45	5.6	1392
59 (Blank)	95	15	2.8	485
60 (Optimum)	81	50	2.3	1587
61 (Optimum)	81	50	2.3	1598

Heating Tests

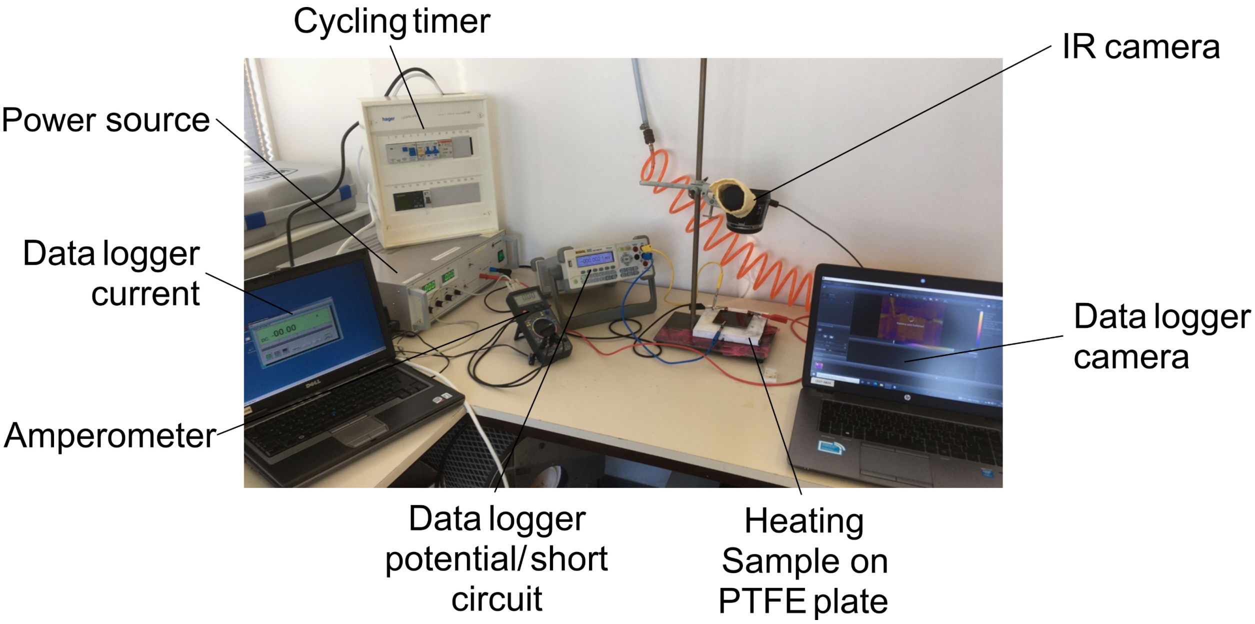

Figure S1: *Heating test setup including the heating sample on an PTFE plate, a power source, an amperometer with data logging, an IR camera with data logging, a voltmeter screening a possible short circuit via the substrate and a timing device to regulate the heating/cooling cycles.*

Water uptake measurement of rGNs-modified epoxy coatings

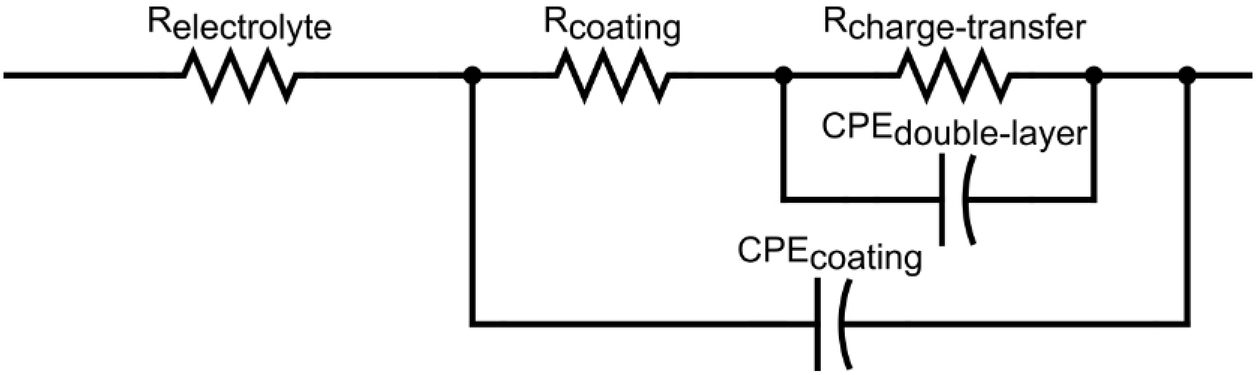

Figure S2: *Equivalent circuit used for fitting of EIS data with resistance of the electrolyte ($R_{electrolyte}$), the resistance of the coating ($R_{coating}$), the constant phase element of the coating ($CPE_{coating}$), the charge-transfer resistance ($R_{Charge-transfer}$) and the double-layer constant phase element ($CPE_{double-layer}$).*

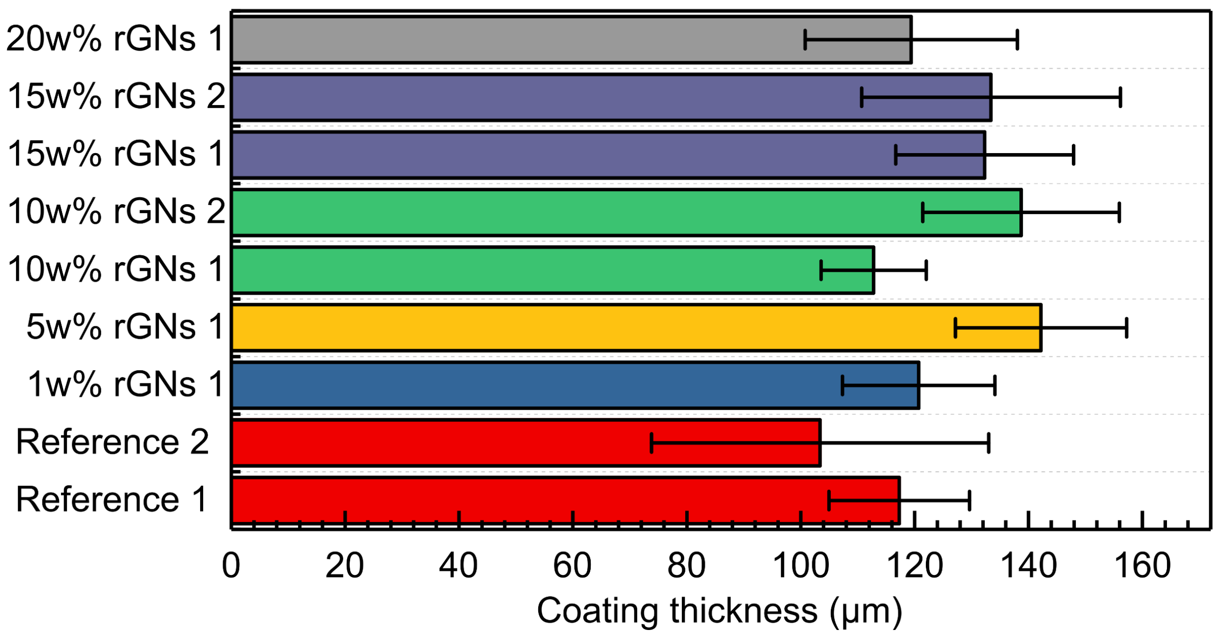

Figure S3: *Epoxy-coating thickness* $d_{coating}$ *of coated Al2024 panels with different rGNs-additive concentrations measured by eddy current method according to DIN EN ISO 2360:2017*[S1].

Characterisation

XRD fitting

To calculate the distribution of layer numbers n, the (002) reflex was fitted with three Gaussian functions. Via Equations S1-S3, the number of layers was calculated as described in our previous work[S2].

$$d = \frac{\lambda}{2 \cdot \sin\theta} \qquad [\text{nm}] \qquad (\text{S1})$$

$$T = \frac{K \cdot \lambda}{\beta \cdot \cos\theta} \qquad [\text{nm}] \qquad (\text{S2})$$

$$n = \frac{T}{d + a} \qquad [1] \qquad (\text{S3})$$

Neutral Salt Spray test

A SaltEvent SC 1000 (Weisstechnik, Germany) was operated according to standard DIN EN ISO 9227:2012 for neutral salt spray test. The edges and uncoated side of the samples were masked with adhesive tape and the coating was scribed centrally with a scratching tool according to van Laer and a tip width of 0.5 mm on a length of 7 cm according to standard ISO 17872. Measurement of corroded area and delaminated area after exposure was carried out using a calliper gauge every 0.5 cm along the scribe according to standard DIN EN ISO 4628-8:2012 Calculation was carried out using Equation S4 for the mean width of delamination $d_{\mathrm{delam.}}$ and Equation S5 for the degree of corrosion c. d_1 describes the mean measured width of delamination, $w_{\mathrm{corrosion}}$ the mean measured width of corrosion and w_{scribe} the width of the scribe.

$$d_{\mathrm{delam.}} = \frac{d_1 - w_{\mathrm{scribe}}}{2} \qquad [\mathrm{mm}] \qquad (\mathrm{S4})$$

$$c = \frac{w_{\mathrm{corrosion}} - w_{\mathrm{scribe}}}{2} \qquad [\mathrm{mm}] \qquad (\mathrm{S5})$$

Results and Discussion

Optimisation of L-Ascorbic acid reduction

Experimental Design

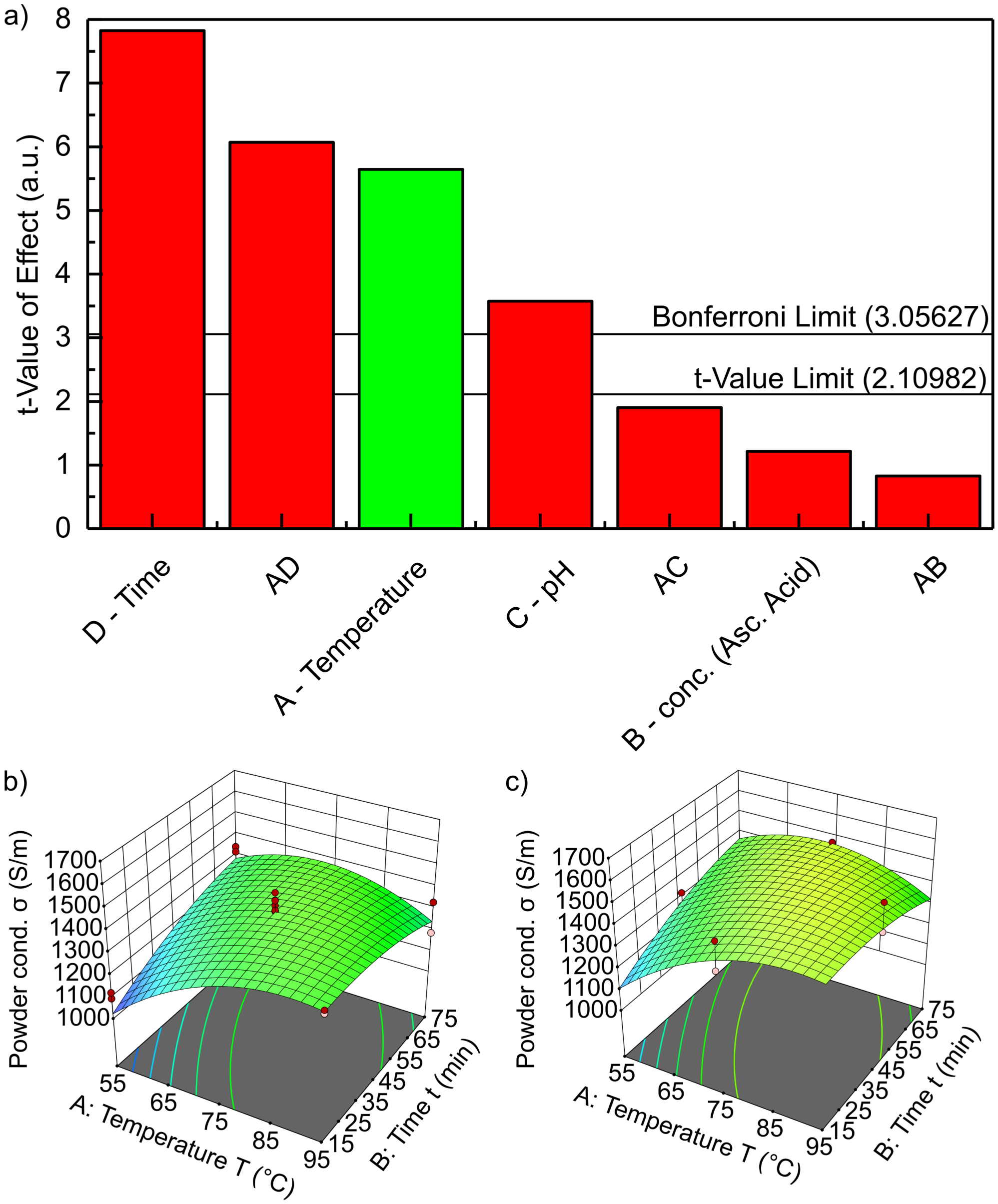

Figure S4: *a) Pareto-Chart of the GNs reduction by L-Ascorbic acid (Screening Design): Parameters are Temperature T (A), concentration of L-Ascorbic acid (B), pH (C), Time t (D) and parameter combinations AB, AC and AD with positive effects (green) and negative effects (red) on the powder conductivity* σ_{powder}*; b-c) Resulting model of rGNs powder conductivity* σ_{powder} *against reduction temperature* T *and time* t *at: b) pH 5.6; c) pH 8.9.*

Table S3: Box-Behnken design of L-Ascorbic acid reduction: Parameter Factors for Cubic Model (σ_{powder} = Intercept + $\sum$ Factor $\cdot$ Parameter) influencing the resulting powder conductivity σ_{powder}

	Powder conductivity factor σ_{powder} [S/m]	p-Value
Intercept	1385.3	
A-Temperature [°C]	72.6	< 0.0001
B-Time [min]	36.3	0.0207
C-V(NH_3 25%) [mL]	-59.0	0.001
AB	-94.5	< 0.0001
AC	not sign.	-
BC	not sign.	-
A^2	-117.8	< 0.0001
B^2	-41.4	0.0088
C^2	143.4	< 0.0001
Model R^2	0.8751	
Lack of fit p-Value	0.2283 (not sign.)	

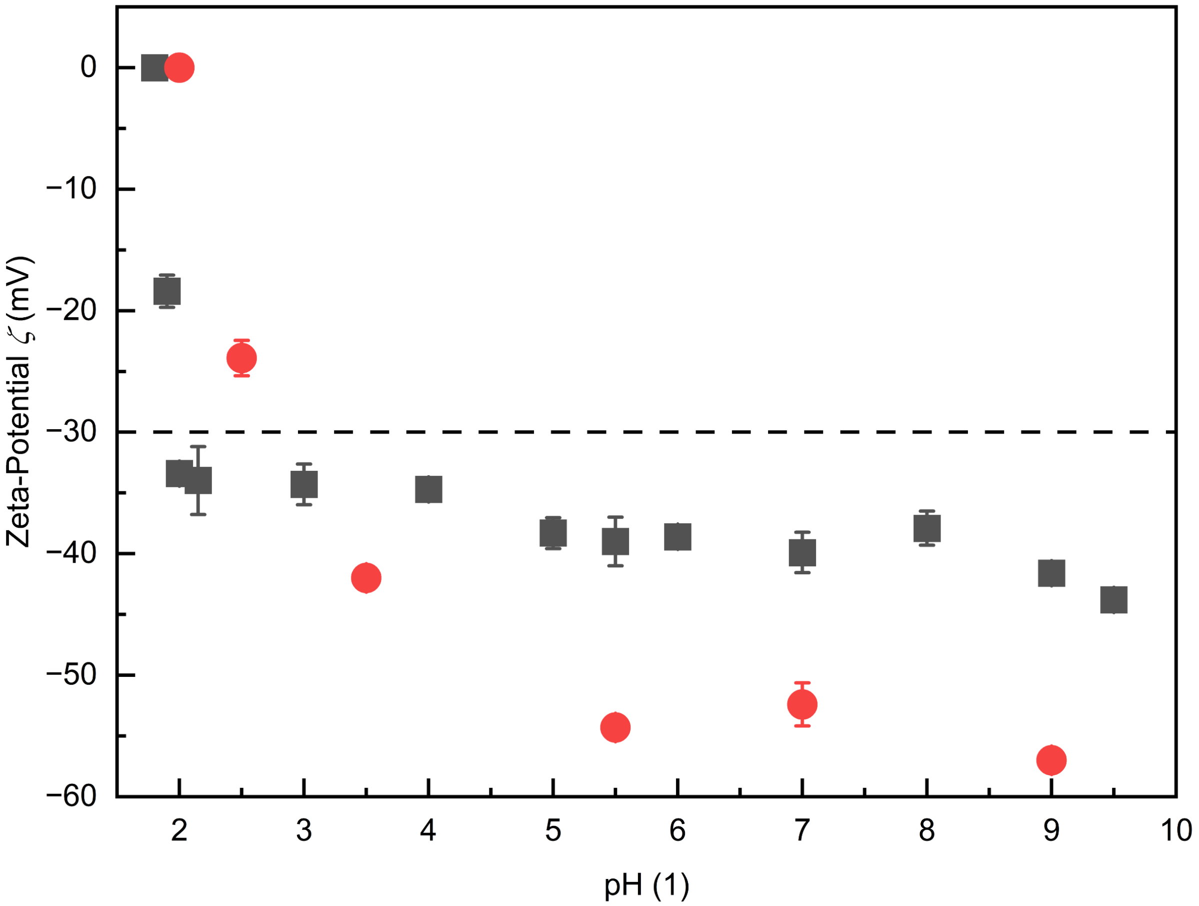

Figure S5: *Zeta Potential of POGNs-dispersions (black) and rGNs-dispersions (red) in water against the pH.*

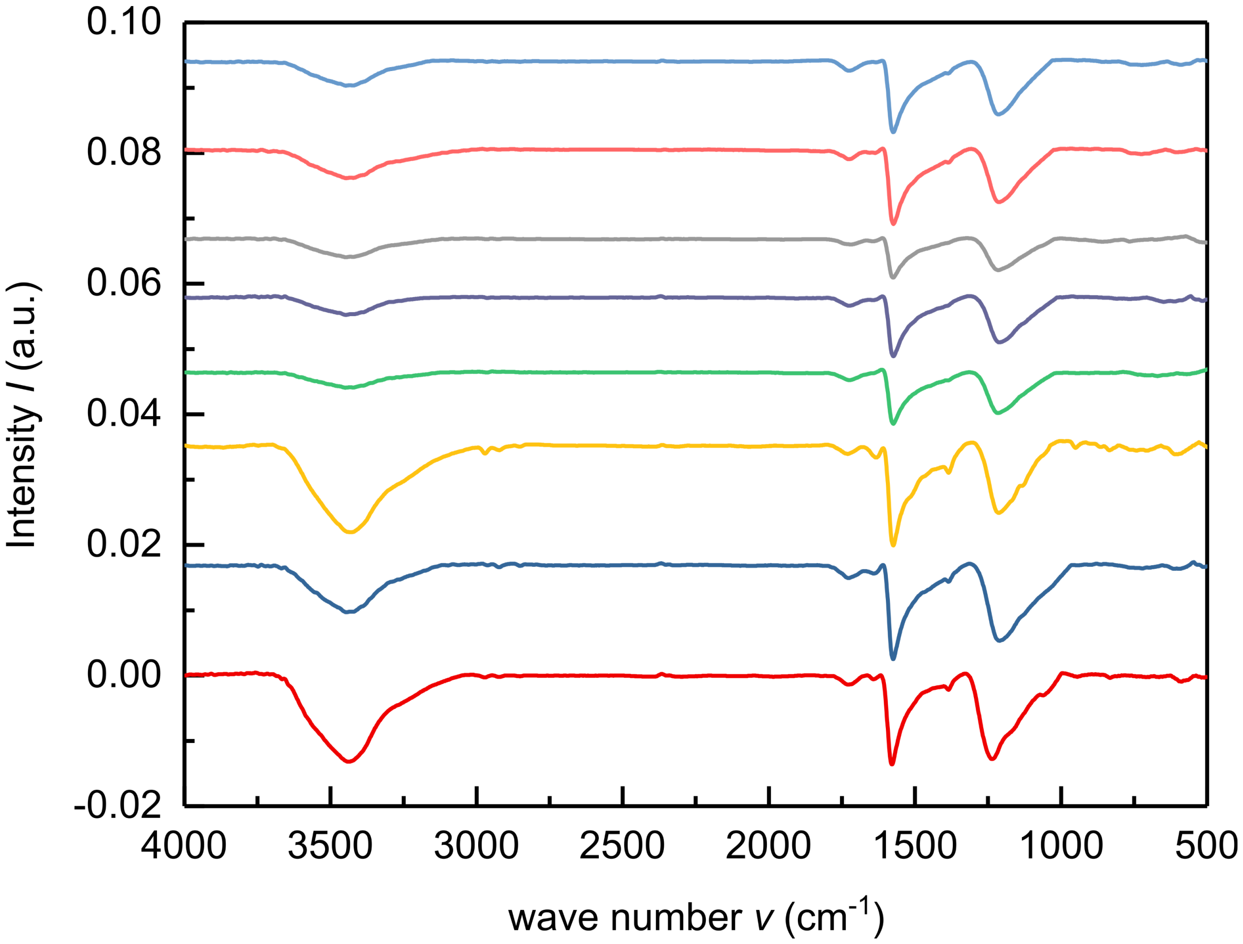

Figure S6: *IR spectra of the POGNs starting material (red) and rGNs samples (Experiment 10, blue; Experiment 17, yellow; Experiment 27, green; Experiment 29, violet; Experiment 32, grey; Experiment 54, pink; Experiment 60, light blue).*

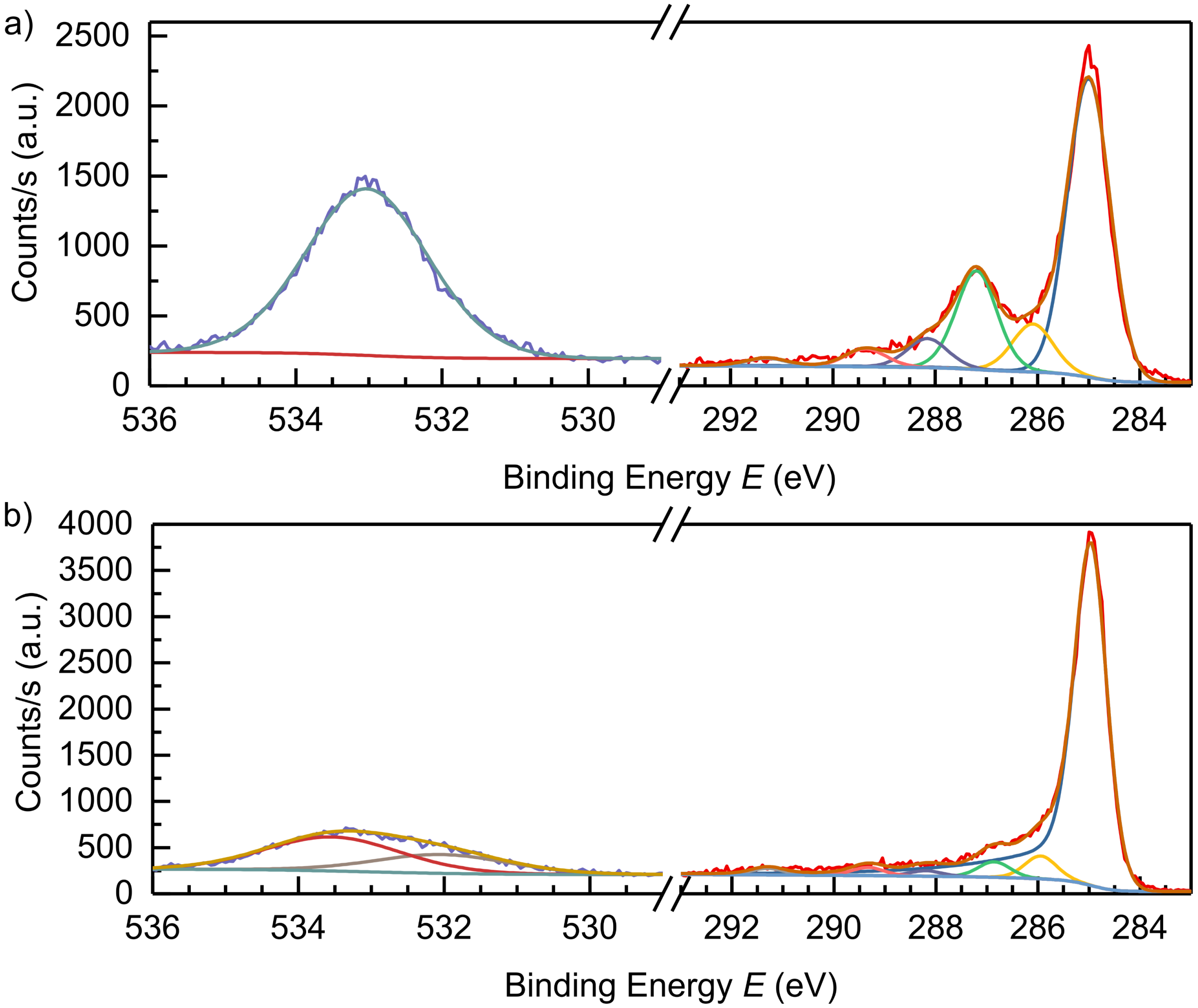

Figure S7: *a) XPS spectra of the electrochemically produced POGNs starting material with the deconvolution of C1s peak (raw data (red), C-C/C-H (blue), C-OH (yellow), C-O-C (green), O-C-O/C=O (violet), O=C-O (grey), pi-pi*(pink)) and O1s peak (raw data (dark blue), fit (turquoise)) as reported previously in*[S2]*; b) XPS spectra of optimised rGNs powder (Experiment 60) with the deconvolution of C1s peak (raw data (red), C-C/C-H (blue), C-OH (yellow), C-O-C (green), O-C-O/C=O (violet), O=C-O (grey), pi-pi*(pink)) and O1s peak (raw data (dark blue), O=C (brown), O-C (dark red)).*

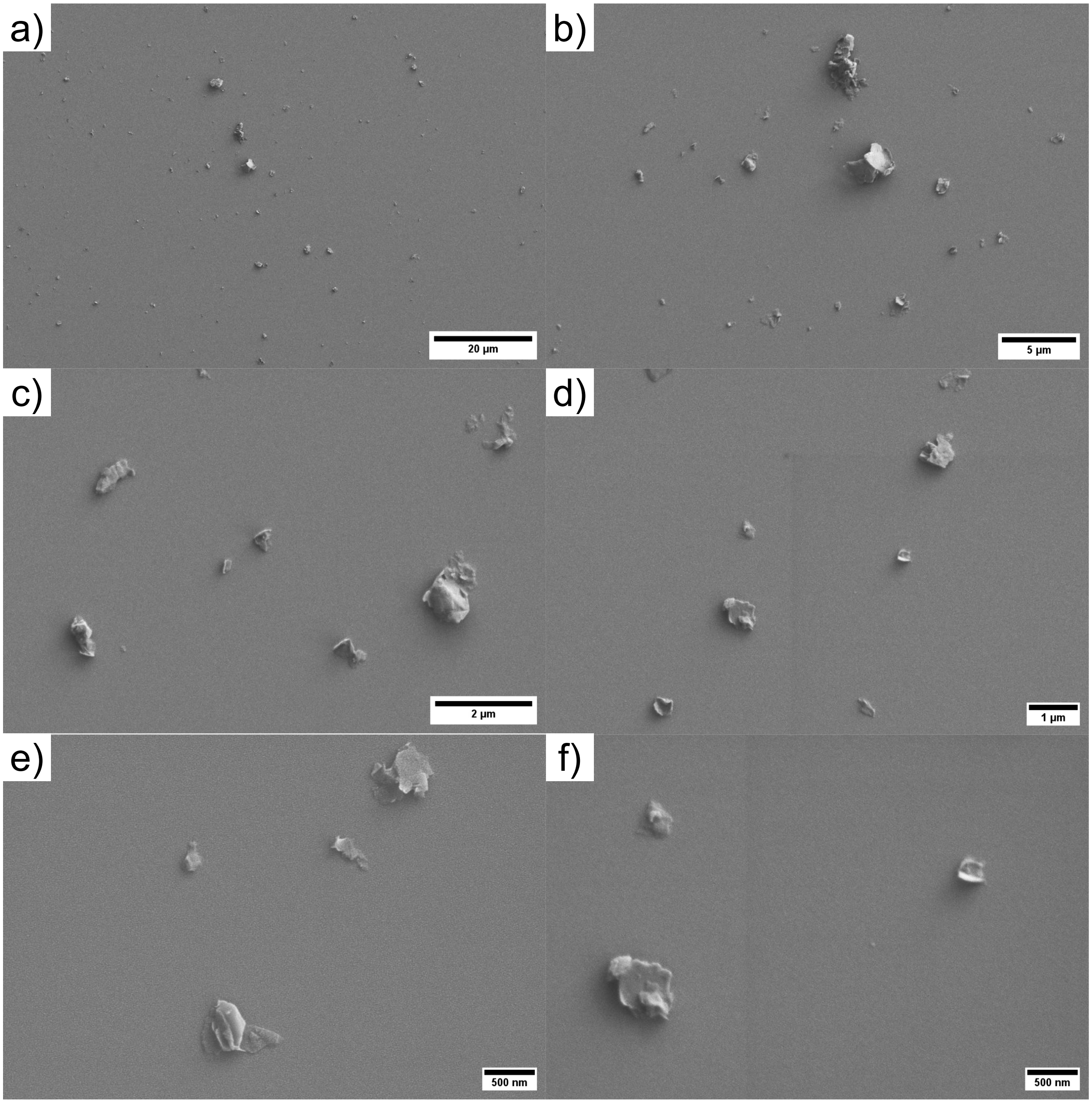

Figure S8: *a-f) SEM micrographs of rGNs flakes spray-coated onto a Si wafer*

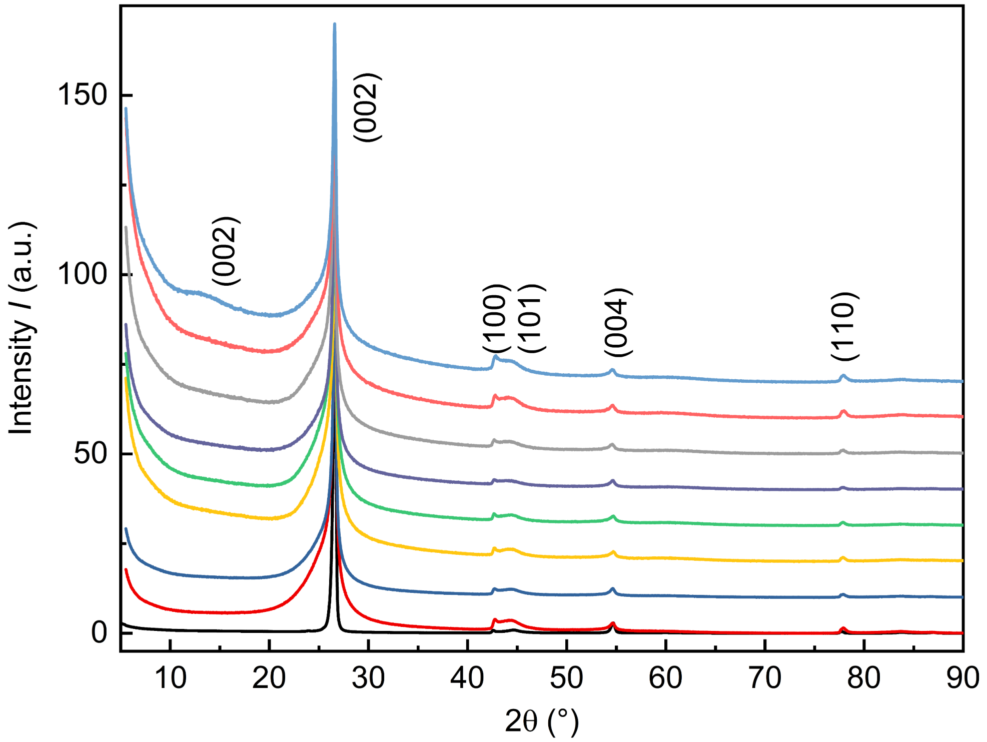

Figure S9: *XRD diffractograms of the graphite used for exfoliation (black), the POGNs starting material (light blue) and rGNs samples (Experiment 10, green; Experiment 17, yellow; Experiment 27, pink; Experiment 29, grey; Experiment 32, violet; Experiment 54, blue; Experiment 60, red).*

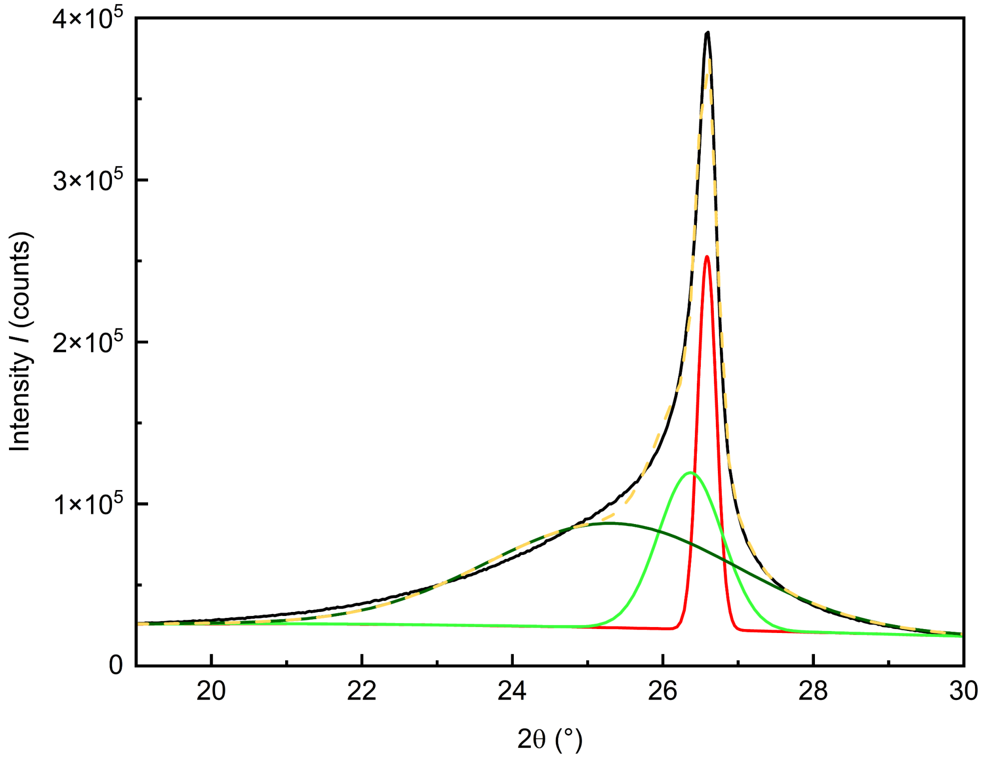

Figure S10: *Optimized rGNs powder (Experiment 60): Fitting of the (002) reflex: measurement data (black), gaussian fit of few-layered material ($n < 10$, dark green), multi-layered material ($10 < n < 25$, light green) and graphitic residues ($n > 25$, red), summary of fits (yellow).*

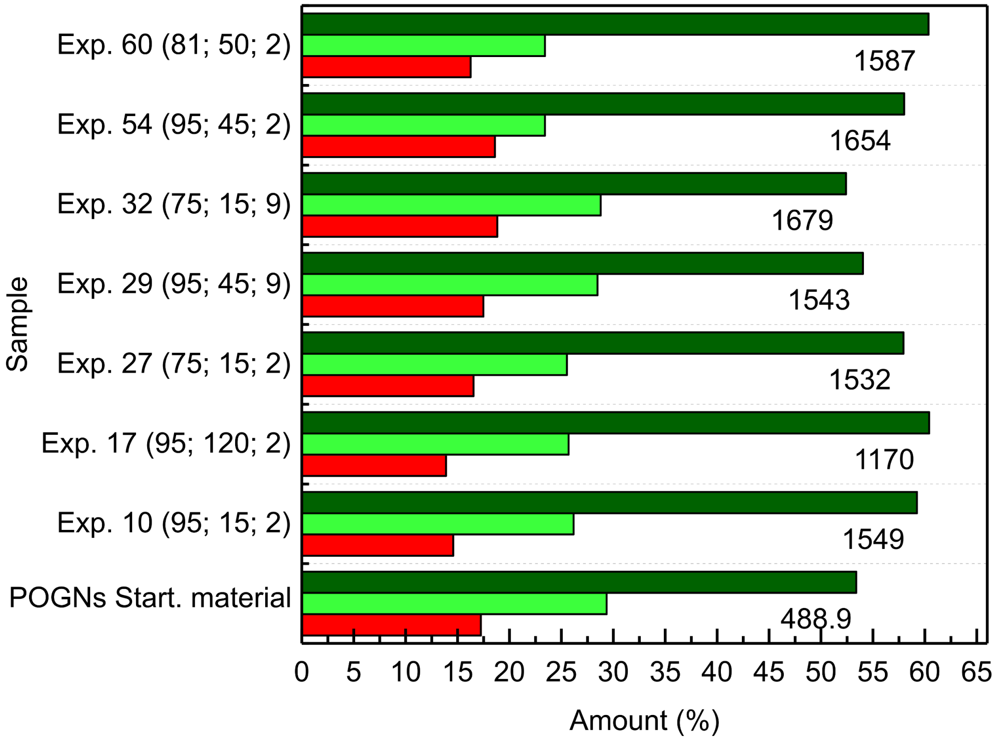

Figure S11: *Calculated Distribution of few-layered material ($n < 10$, dark green), multi-layered material ($10 < n < 25$, light green) and graphitic residues ($n > 25$, red) via fitting of the (002) reflex; the labels refer to the powder conductivity σ_{powder} in S/m.*

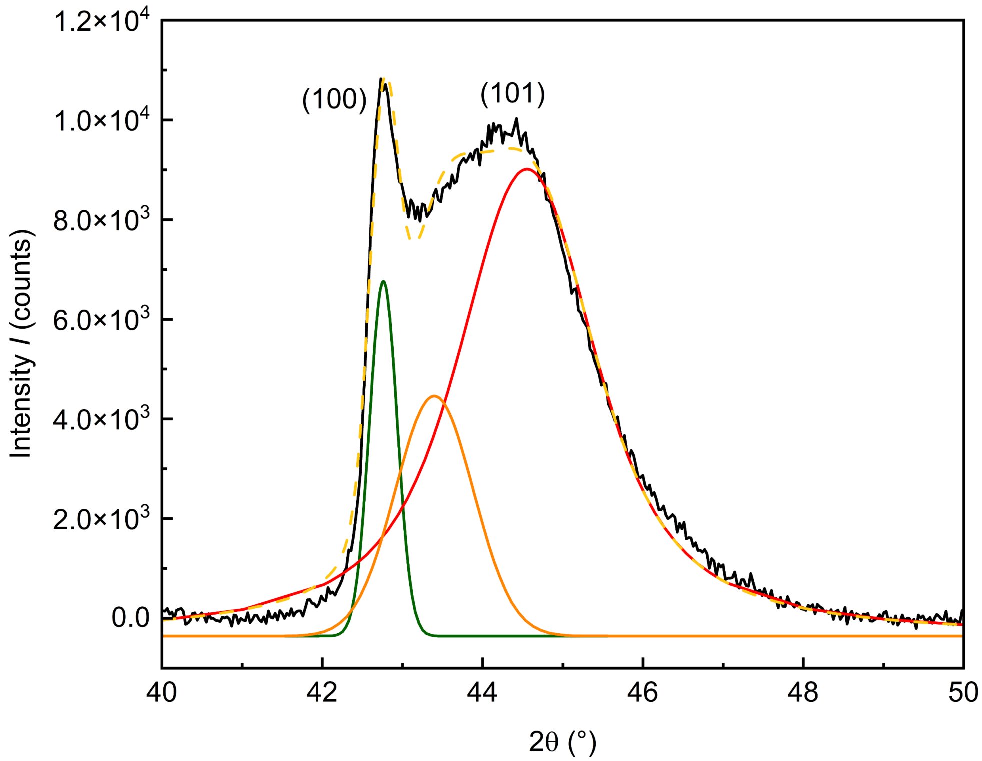

Figure S12: *Optimized rGNs powder (Experiment 60): Fitting of the (100) (dark green) and (101) (orange and red) reflexes and summary of fits (yellow).*

Table S4: Crystallite sizes according to Rietveld analysis of graphite, POGNs starting material and optimized rGNs

	Peakposition (002) [°]	FWHM (002) [°]	$t_{Crystallite}$ [nm]	Peakposition (100) [°]	FWHM (100) [°]	$d_{Crystallite}$ [nm]
Graphite	26.57	0.32	32.51	42.62	0.33	30.83
POGNs starting material	25.39	3.14	2.52	42.77	0.43	22.12
rGNs	25.33	3.91	2.02	42.76	0.41	23.64

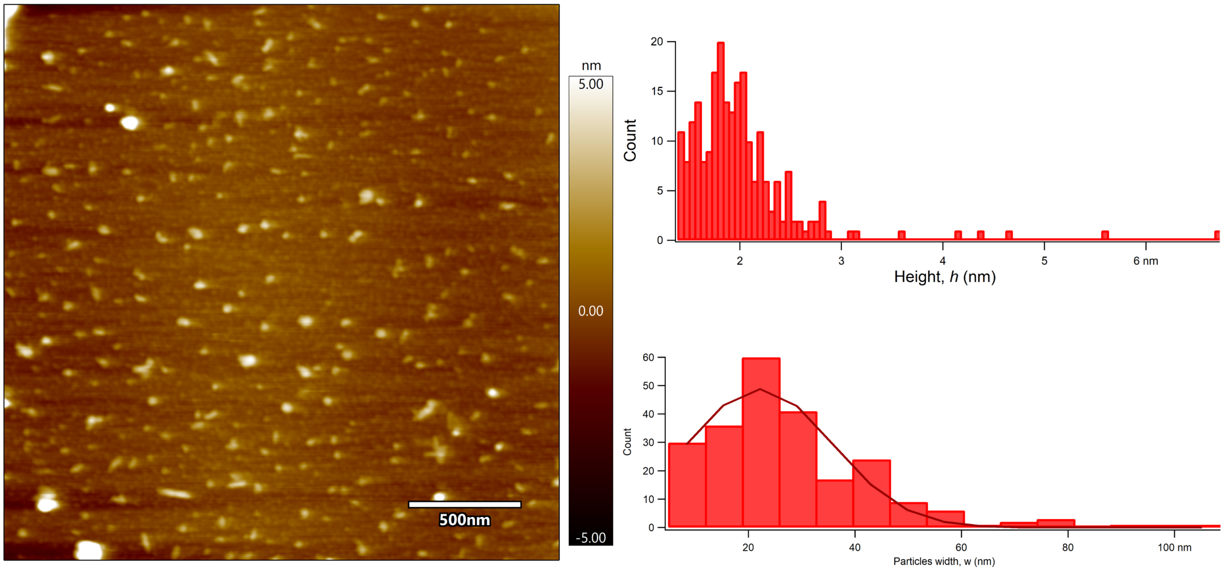

Figure S13: *AFM topography of rGNs particles on a Mica after extensive ultrasonication. Scan size is* $2.5 \times 2.5\,\mu m^2$*. On the right side, on top and bottom height and width distribution respectively.*

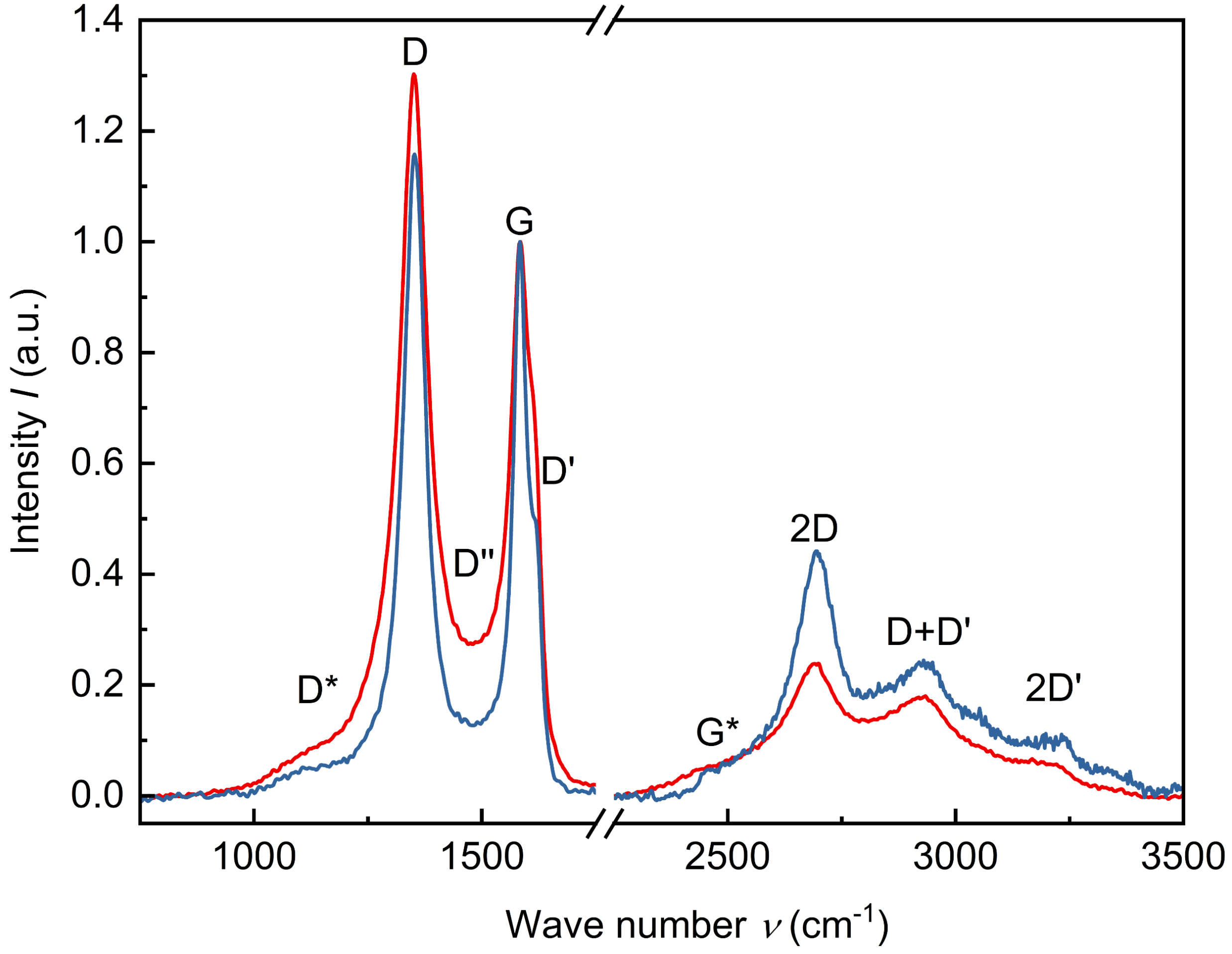

Figure S14: *Raman Spectra of the graphene oxide starting material (red) and the reduced graphene oxide after optimization (blue).*

rGNs-based electrothermal De-Icing layer

Roughness measurement

Table S5: Roughness data of epoxy-coated Al2024 and glass fibre composite substrate

Substrate	Epoxy-coated Al2024	GFC
R_a [µm]	0.02 ± 0.01	5.87 ± 1.29
R_z [µm]	0.25 ± 0.20	28.0 ± 6.9
Peaks on 0.8 mm measurement length n_{Peaks} [1]	—	10.5 ± 1.2
Increased length on 5cm width due to roughness [cm]	—	3.7 ± 0.9
Roughness-related factor r [1]	1	1.73 ± 0.18

Monte-Carlo Simulation

To determine the contact area of randomly applied particles on a surface, Monte-Carlo Simulations were executed using Geogebra Classic 6 (Geogebra GmbH, Austria, geogebra.org). The rGNs spray-coating was simulated with a number of particles $n_{crystallites}$ (100-2000) with a diameter of 23.64 nm, whereby the individual crystallitic structures were simplified as circles (example shown in Figure S15 a)). The crystallites were randomly distributed within a test area of 1x1µm. A random pattern of 10^5 Monte-Carlo points m is set into the test area. The contact area A_{cont} is calculated by the ratio of the number of Monte-Carlo points within a contact area m_{cont} and the total number of points m (shown in Equation S6). By normalizing A_{cont} to the number of crystallites $n_{crystallites}$, the effective contact Area per Flake $A_{cont/Crystallite}$ is calculated (Equation S7). Figure S15 b) shows $A_{cont/Crystallite}$ depicted against the number of crystallites $n_{crystallites}$. A linear fit is used to determine the contact area increase per crystallite $\Delta A_{cont/Crystallite}$ with increasing number of particles.

$$A_{cont} = \frac{m_{cont}}{m} \qquad [\mu m^2] \qquad (S6)$$

$$A_{cont/crystallite} = \frac{A_{cont}}{n_{crystallites}} \qquad [\mu m^2] \qquad (S7)$$

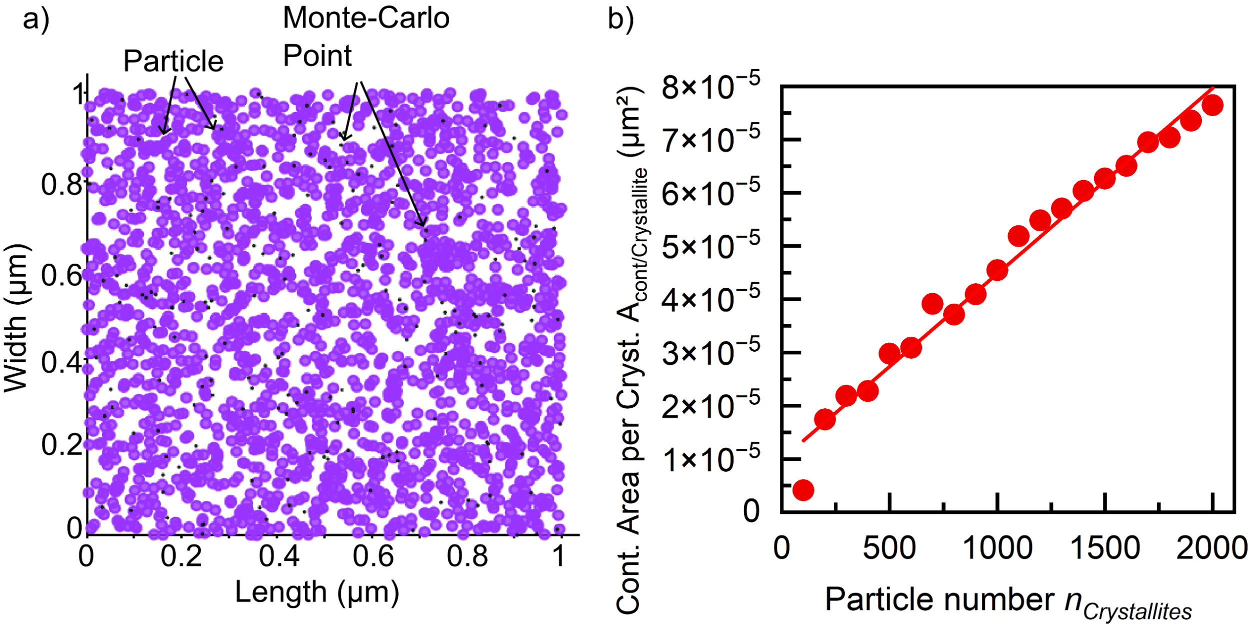

Figure S15: *a) Example of Monte-Carlo Simulation to determine the contact area of randomly distributed spray-coated crystallitic structures (purple, here: 2000) with Monte-Carlo Points (black, here: 200), b) Contact area per particle* $A_{cont/crystallite}$ *against the particle number* $n_{crystallites}$

Model factors

Table S6: Factors influencing the relation between sheet resistance R_{heat} and mass rGNs per area m_{rGNs} (see Equation 9).

**rGNs powder-dependant Factor**	**Value**	**determined by**
Powder conductivity σ_{powder} [S/m]	1593	Powder conductivity measurement (see Figure 1 b)
Lateral Crystallite area $A_{Crystallite}$ [nm^2]	558.67	Crystallite size according to fitting of the (100)-Reflex in XRD (see Table S4)
Crystallite thickness $t_{Crystallite}$ [nm]	2.02	Crystallite size of few-layered part according to fitting of the (002)-Reflex in XRD (see Table S4)
Crystallite volume $V_{Crystallite}$ [nm^3]	$1.13 \cdot 10^3$	Calculated from Crystallite diameter and thickness
Krenchel Orientation Factor η_0 [1]	0.53	according to Papageorgiou et al.[S3]
Density ρ_{powder} [g/cm^3]	1.91	according to Graphenea Inc.[S4]
Increase of contact area per Crystallite $\Delta A_{cont/crystallite}$ [µm^2]	$3.48 \cdot 10^{-8}$	Monte-Carlo Simulations of particles (see Figure S15 in the SI)
Heating layer thickness t_{layer} [µm]	28	Cross-Cut analysis (see Figure 3 c)-d))
**Substrate-dependant Factor**	**Value** ◦**Epoxy-coated Al2024** ◦**GFC**	**determined by**
Roughness-Factor r [1]	◦1.0 ◦1.734	Surface roughness measurement (see Equation 6 & Table S5 in the SI)

Cost calculation of rGNs production

The following cost calculation of the rGNs includes costs for material and electricity on lab scale production. Table S7 summarizes the required energy and related costs (price: 0.2454 €/kWh) for the production of 1 kg rGNs.

Table S7: Energy Requirement per kg rGNs production and considered costs at a price of 0.2454 €/kWh

Energy Exfoliation [kWh]	60.8
Energy Ultrasonication [kWh]	21.6
Energy Filtration [kWh]	38.4
Energy Drying [kWh]	14.4
Energy Reduction [kWh]	7.8
**Total Energy Requirement [kWh]**	**135.2**
**Total Energy Costs [€]**	**33.2**

Table S8 summarizes the material and energy cost required for the production of 1 kg rGNs at lab scale (35 g batches). Material prices were calculated according to current supplier prices (January 2023) as mentioned in the Materials section. Additional costs related to spray-coating include the costs for solvent and ultrasonication per m^2 De-Icing layer considering no recovery of solvent after evaporation.

Table S8: Material and energy costs per kg rGNs production

Cost Graphite Rods [€]	284.0
Cost NaOH Pellets [€]	30.6
Cost H_2SO_4 96% [€]	33.0
Cost Deionised Water [€]	10.1
Cost L-Ascorbic Acid [€]	134.8
Cost Energy [€]	33.2
**Total Costs per kg rGNs [€]**	**527.6**
**Resulting rGNs costs per m^2 at 2 mg/cm^2 and 30 % Overspray [€]**	**15.1**
Additional Costs related to Spray-Coating (n-Butyl acetate, Ultrasonication) [€]	4.4

De-Icing functionality

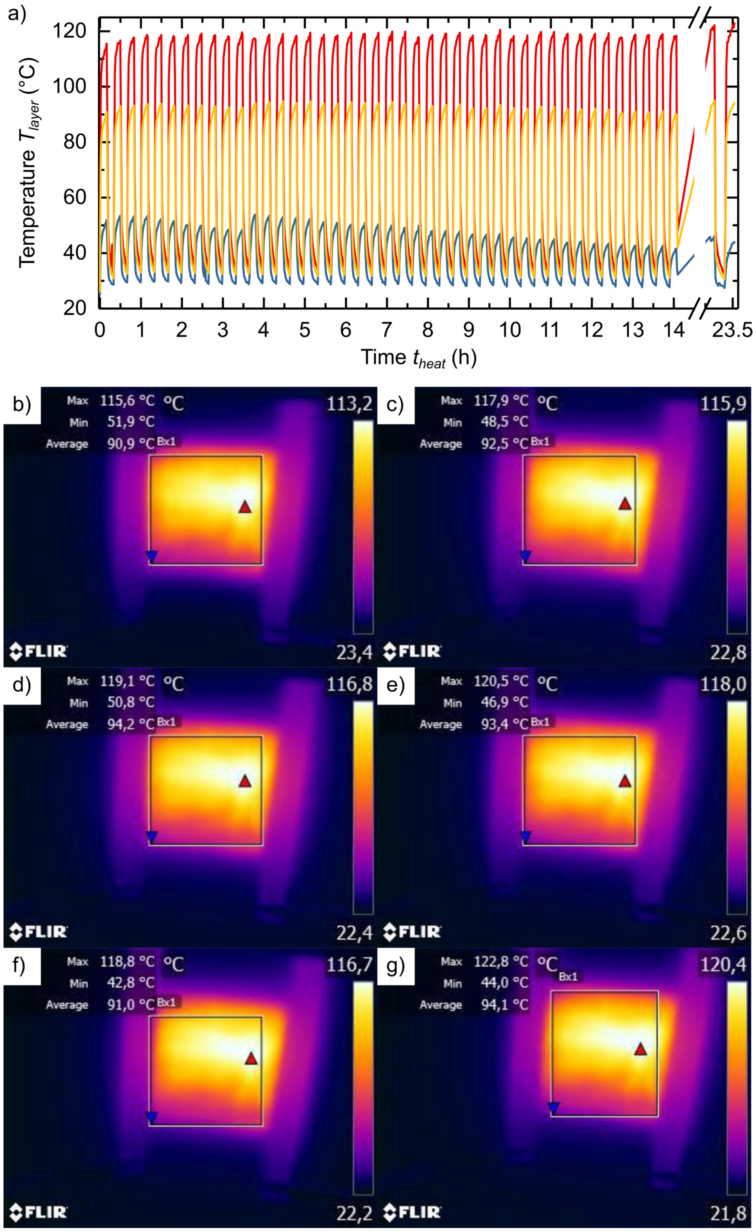

Figure S16: *a) Detailed temperature profile of the heating cycles 1-43 and 70-71 with the maximum (red), average (yellow) and minimum (blue) temperature of the heating layer; b-g) Thermal image of the De-Icing sample after 10 min of heating at the: b) 1st, c) 10th, d) 20th, e) 30th, f) 40th and g) 71st cycle.*

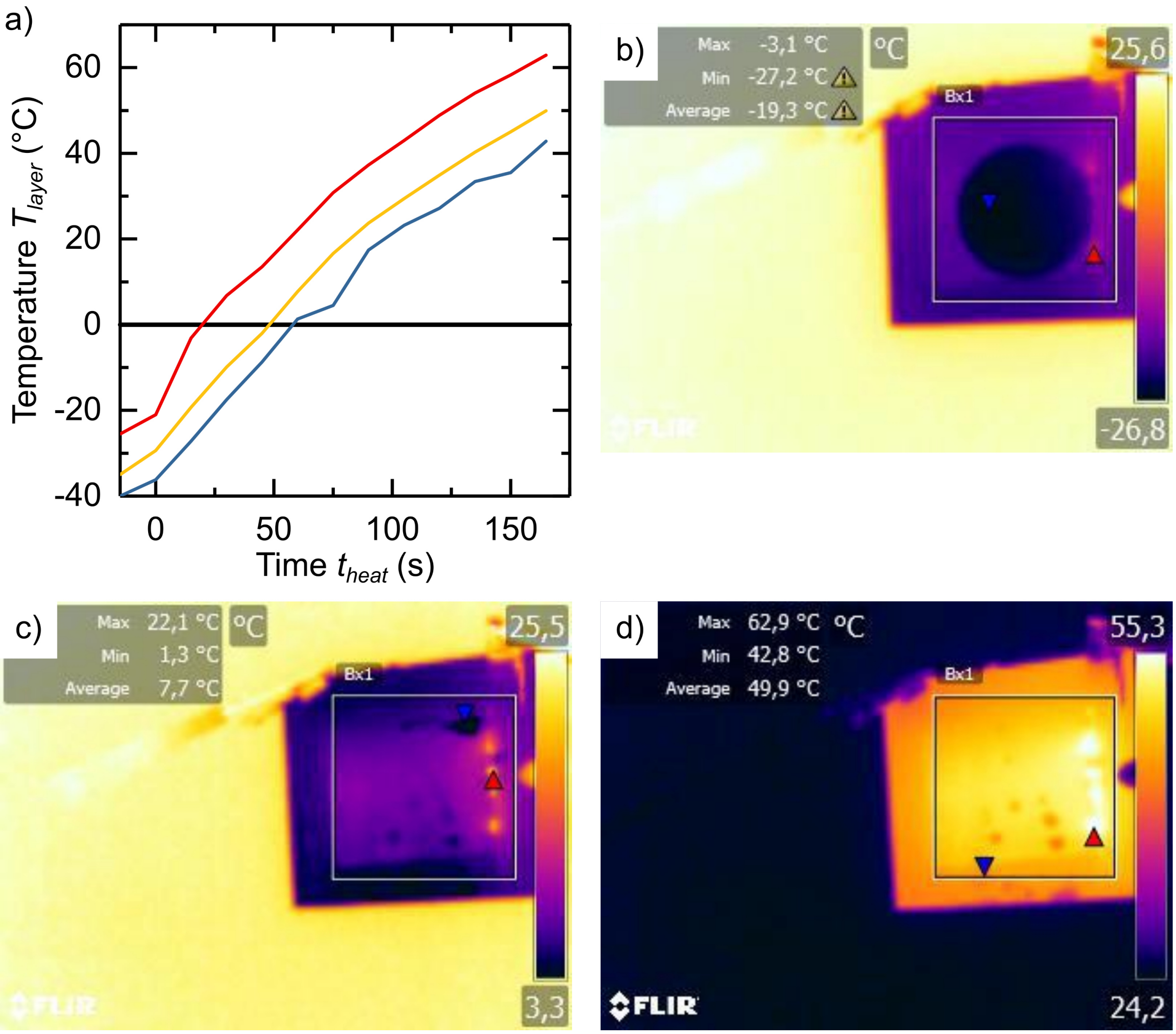

Figure S17: *Heating test with Ice pellet on surface: a) Detailed temperature profile of the heating layer with the maximum (red), average (yellow) and minimum (blue) temperature; b-d) Thermal image after b) 15 s; c) 60 s; d) 165 s.*

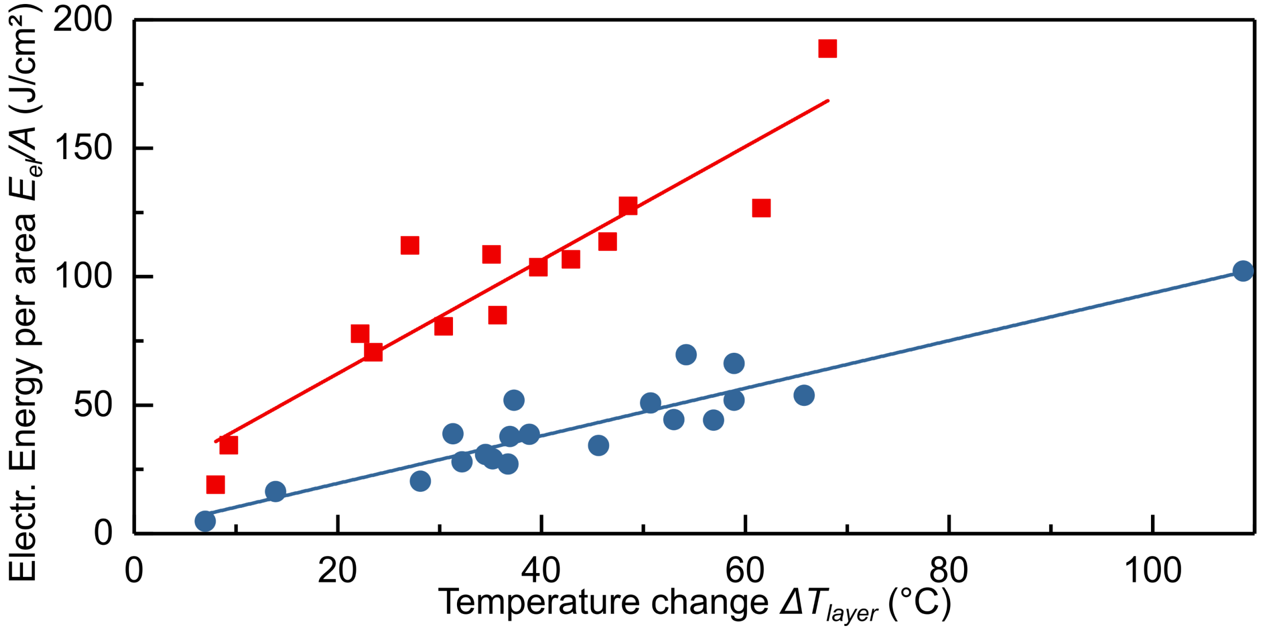

Figure S18: *Applied electrical energy per area E_{el}/A against achieved temperature change ΔT_{layer} during various heating tests on epoxy-coated Al2024 (red) and glass fibre composite (blue) substrate with corresponding linear fits*

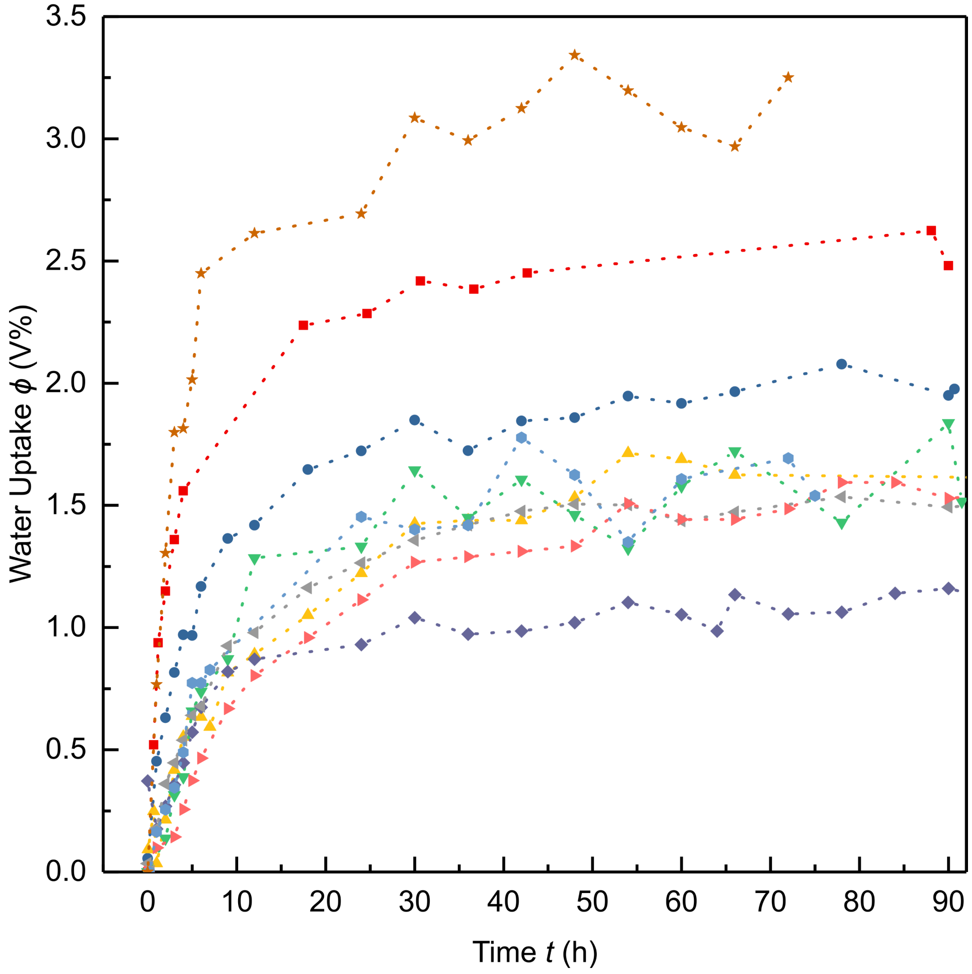

Figure S19: *Water uptake ϕ against time t of different epoxy coatings: Reference 1 (red), Reference 2 (blue), 1 w% rGNs 1 (yellow), 5 w% rGNs 1 (green), 10 w% rGNs 1 (violet), 10 w% rGNs 2 (grey), 15 w% rGNs 1 (pink), 15 w% rGNs 2 (light blue), 20 w% rGNs 1 (brown).*

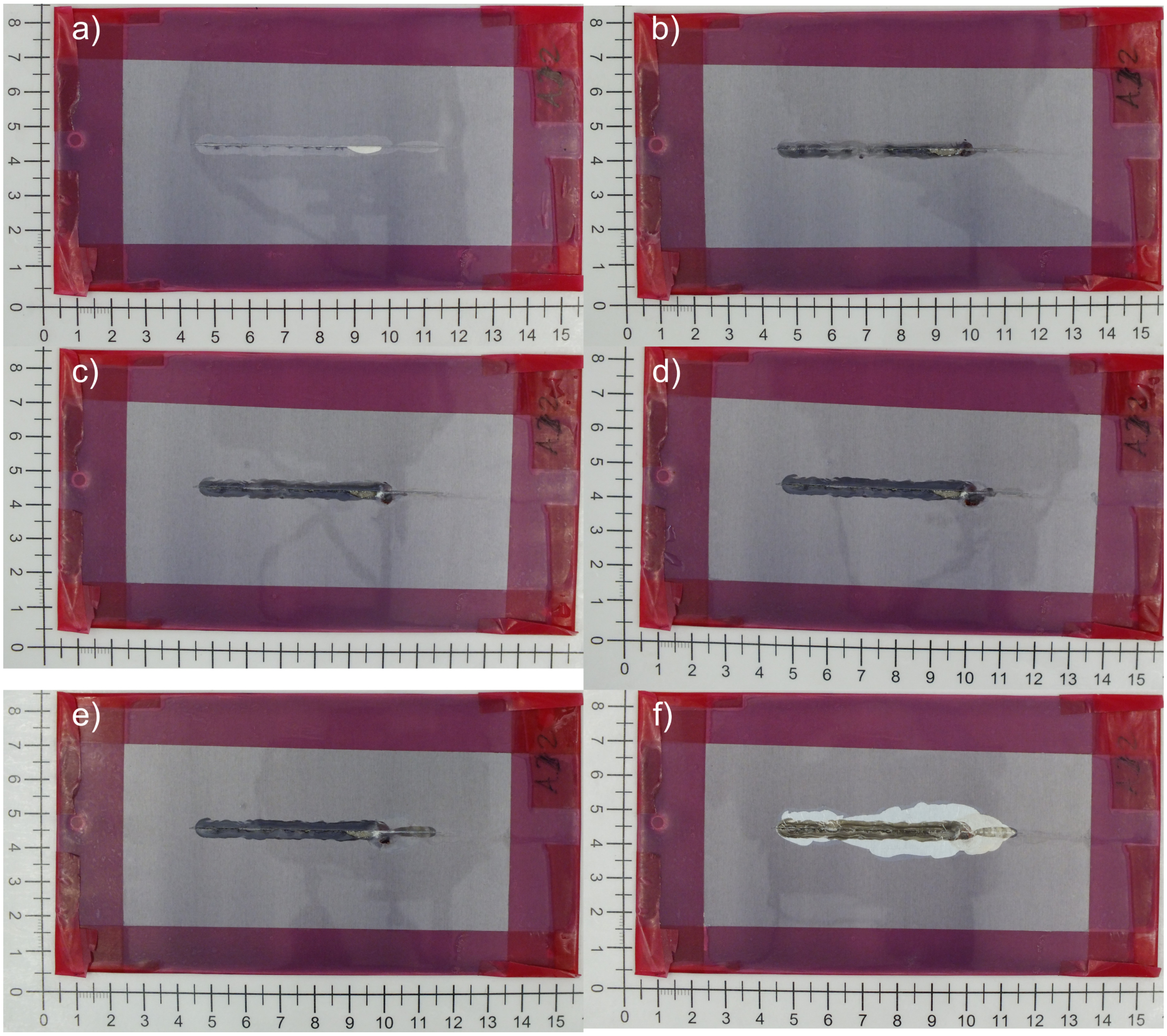

Figure S20: *Neutral salt spray test epoxy-coated reference sample after: a) 0 h; b) 516 h; c) 1018 h; d) 1450 h; e) 2026 h of exposition and f) after 2026 h exposition and removal of the delaminated area.*

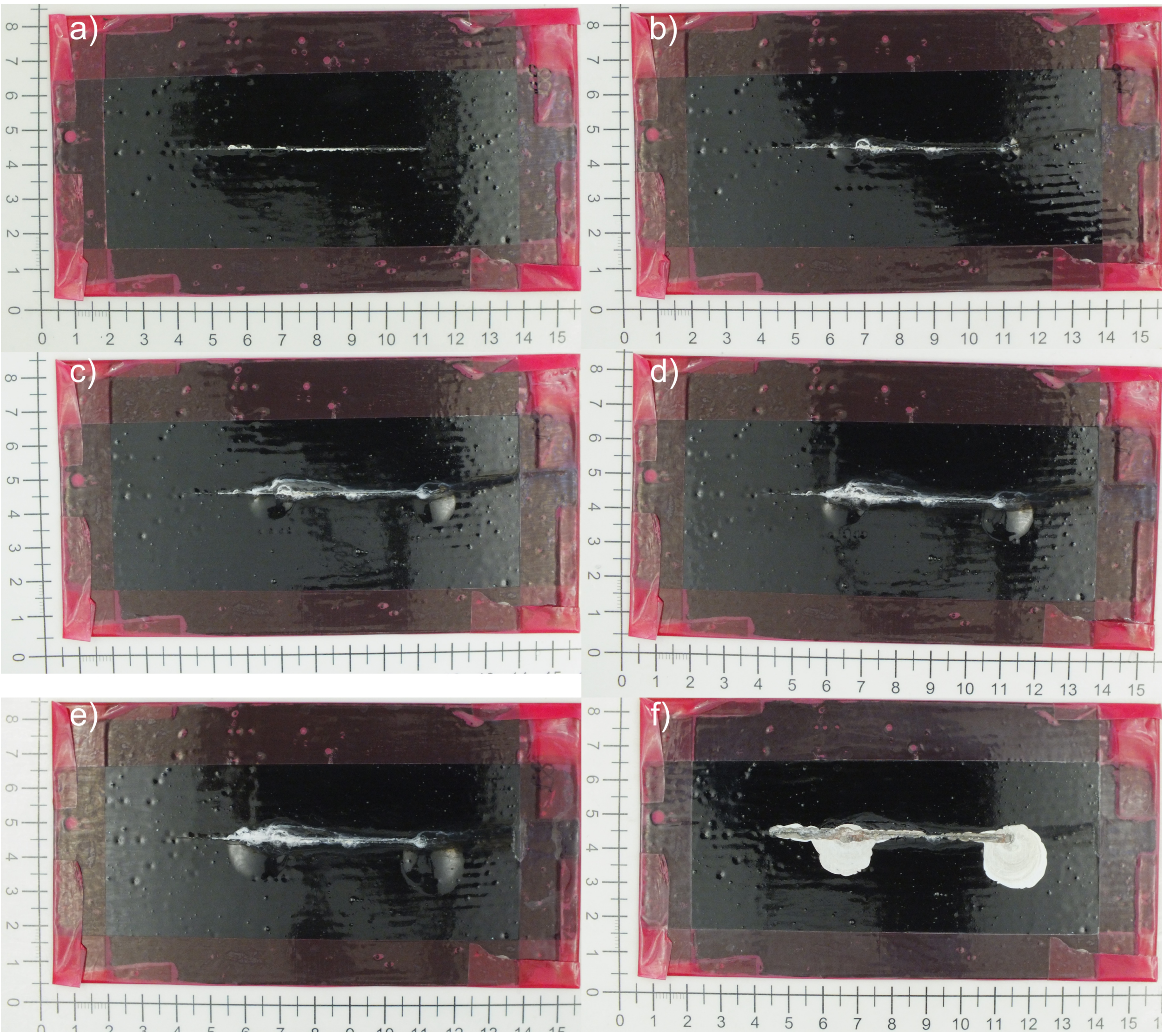

Figure S21: *Neutral salt spray test epoxy-coated sample with 10 w% rGNs additive after: a) 0 h; b) 516 h; c) 1018 h; d) 1450 h; e) 2026 h of exposition and f) after 2026 h exposition and removal of the delaminated area.*

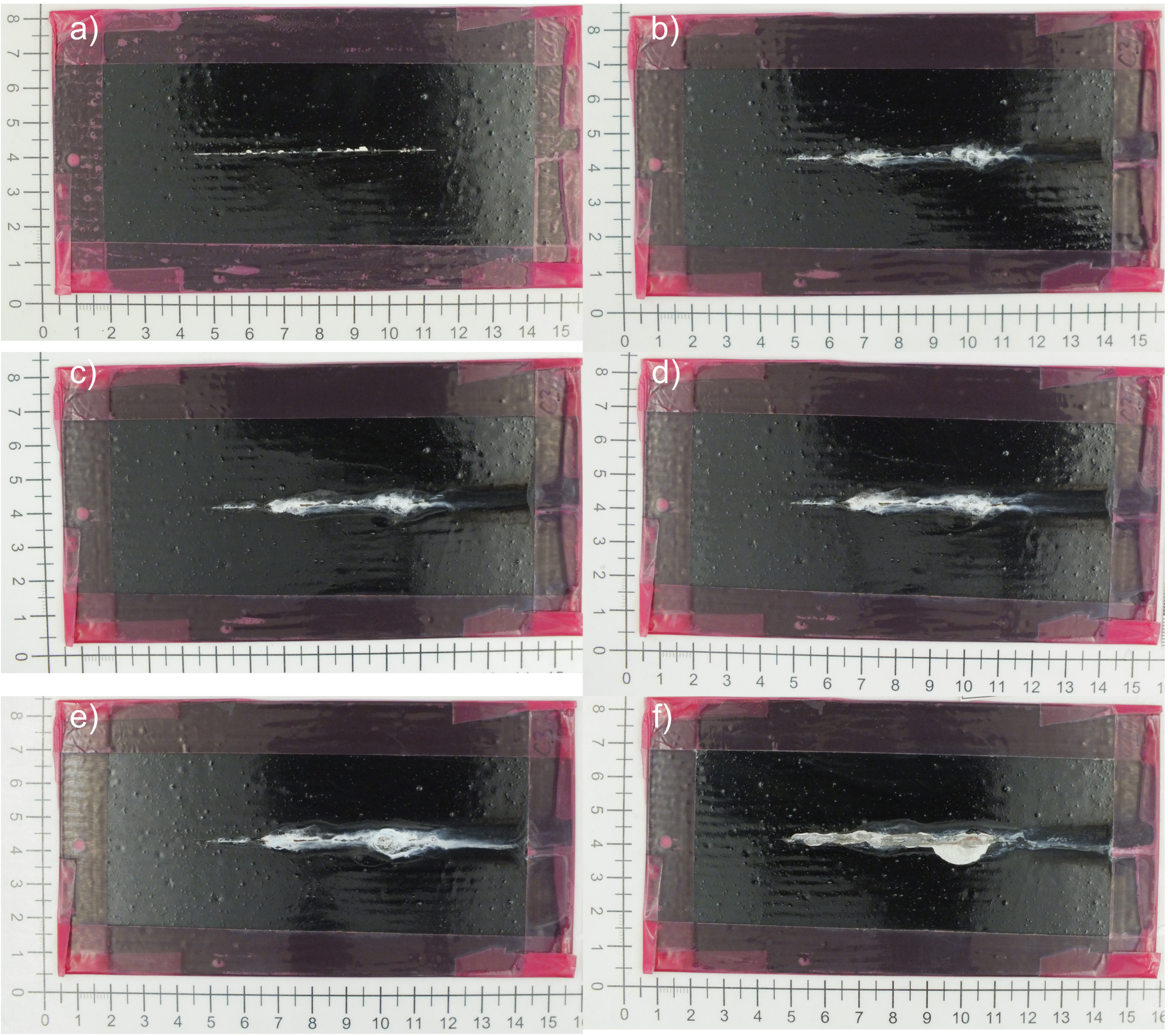

Figure S22: *Neutral salt spray test epoxy-coated sample with 15 w% rGNs additive after: a) 0 h; b) 516 h; c) 1018 h; d) 1450 h; e) 2026 h of exposition and f) after 2026 h exposition and removal of the delaminated area.*

References

(S1) ISO Central Secretary, *DIN EN ISO 2360:2017, Non-conductive coatings on non-magnetic electrically conductive base metals - Measurement of coating thickness - Amplitude-sensitive eddy-current method*; Beuth Verlag: Geneva, CH, 2017.

(S2) Ostermann, M.; Velicsanyi, P.; Bilotto, P.; Schodl, J.; Nadlinger, M.; Fafilek, G.;

Lieberzeit, P. A.; Valtiner, M. Development and Up-Scaling of Electrochemical Production and Mild Thermal Reduction of Graphene Oxide. *Materials* **2022**, *15*, 4639.

(S3) Papageorgiou, D. G.; Li, Z.; Liu, M.; Kinloch, I. A.; Young, R. J. Mechanisms of mechanical reinforcement by graphene and carbon nanotubes in polymer nanocomposites. *Nanoscale* **2020**, *12*, 2228–2267.

(S4) AZoNano and Graphenea Inc., Reduced graphene oxide: Properties, applications and production methods. 2015; `https://www.azonano.com/article.aspx?ArticleID=4041#:~:text=Density\%3A\%201.91g\%2Fcm&text=Dispersability\%3A\%20It\%20can\%20be\%20dispersed,\%3A\%20666\%2C7\%20S\%2Fm`, Last accessed: 2022-10-17.
